# Interleukin-7 and interleukin-15 drive CD4^+^CD28^null^ T lymphocyte expansion and function in patients with acute coronary syndrome

**DOI:** 10.1093/cvr/cvaa202

**Published:** 2020-07-09

**Authors:** Jessica Bullenkamp, Veronica Mengoni, Satdip Kaur, Ismita Chhetri, Paraskevi Dimou, Zoë M J Astroulakis, Juan Carlos Kaski, Ingrid E Dumitriu

**Affiliations:** 1 Molecular and Clinical Sciences Research Institute, St. George’s, University of London, Cranmer Terrace, London SW17 0RE, UK; 2 Cardiology Clinical Academic Group, St George’s University Hospitals NHS Foundation Trust, Blackshaw Road, London SW17 0QT, UK

**Keywords:** Atherosclerosis, Inflammation, T lymphocytes, CD28^null^ T cells, Cytokines

## Abstract

**Aims:**

Inflammation has important roles in atherosclerosis. CD4^+^CD28^null^ (CD28^null^) T cells are a specialized T lymphocyte subset that produce inflammatory cytokines and cytotoxic molecules. CD28^null^ T cells expand preferentially in patients with acute coronary syndrome (ACS) rather than stable angina and are barely detectable in healthy subjects. Importantly, ACS patients with CD28^null^ T-cell expansion have increased risk for recurrent acute coronary events and poor prognosis, compared to ACS patients in whom this cell subset does not expand. The mechanisms regulating CD28^null^ T-cell expansion in ACS remain elusive. We therefore investigated the role of cytokines in CD28^null^ T-cell expansion in ACS.

**Methods and results:**

High-purity sorted CD4^+^ T cells from ACS patients were treated with a panel of cytokines (TNF-α, IL-1β, IL-6, IL-7, and IL-15), and effects on the number, phenotype, and function of CD28^null^ T cells were analysed and compared to the control counterpart CD28^+^ T-cell subset. IL-7- and IL-15-induced expansion of CD28^null^ T cells from ACS patients, while inflammatory cytokines TNF-α, IL-1β, and IL-6 did not. The mechanisms underlying CD28^null^ T-cell expansion by IL-7/IL-15 were preferential activation and proliferation of CD28^null^ T cells compared to control CD28^+^ T cells. Additionally, IL-7/IL-15 markedly augmented CD28^null^ T-cell cytotoxic function and interferon-γ production. Further mechanistic analyses revealed differences in baseline expression of component chains of IL-7/IL-15 receptors (CD127 and CD122) and increased baseline STAT5 phosphorylation in CD28^null^ T cells from ACS patients compared to the control CD28^+^ T-cell subset. Notably, we demonstrate that CD28^null^ T-cell expansion was significantly inhibited by Tofacitinib, a selective JAK1/JAK3 inhibitor that blocks IL-7/IL-15 signalling.

**Conclusion:**

Our novel data show that IL-7 and IL-15 drive the expansion and function of CD28^null^ T cells from ACS patients suggesting that IL-7/IL-15 blockade may prevent expansion of these cells and improve patient outcomes.

## Graphical Abstract

**Figure:**
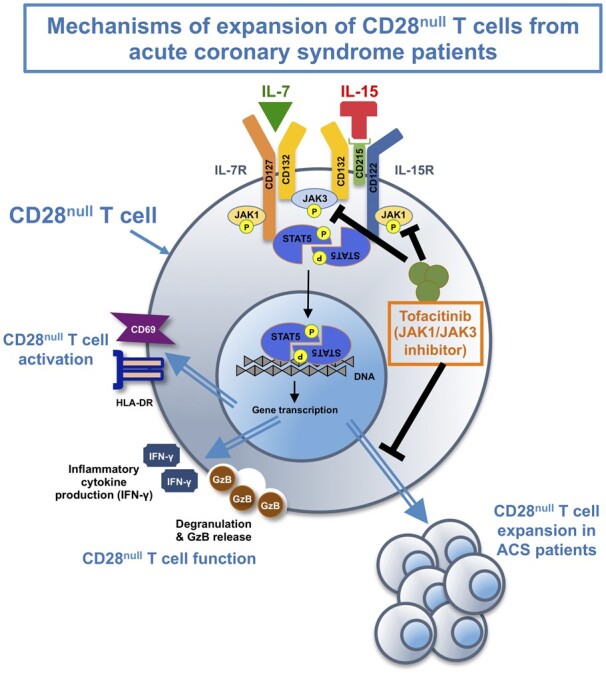

## 1. Introduction

Coronary artery disease (CAD) and acute coronary syndrome (ACS) remain a major cause of death and morbidity worldwide despite considerable advances in diagnosis, prevention, and treatment.^1^ T lymphocytes have pivotal roles in atherosclerosis and pathogenesis of CAD.^2^^,^^3^ CD4^+^CD28^null^ (CD28^null^) T cells are an inflammatory subset of T lymphocytes defined by the lack of the co-stimulatory receptor CD28. CD28^null^ T cells are unique as they are not present in mice but have been identified exclusively in humans with chronic inflammatory diseases.^4^^,^^5^ These cells preferentially expand in the circulation and atherosclerotic plaques of patients with ACS rather than stable angina,^6^^,^^7^ and are nearly undetectable in healthy individuals.^4^^,^^8^ Importantly, ACS patients with expansion of CD28^null^ T cells have increased risk for recurrent acute coronary events and poor prognosis compared to ACS patients in whom this subset does not expand.^9^ Moreover, increased CD28^null^ T cells are an independent predictor of future acute coronary events in ACS patients.^9^ We have previously demonstrated that in contrast to the conventional control CD4^+^CD28^+^ (CD28^+^) T-cell subset, CD28^null^ T cells from ACS patients produce high levels of inflammatory cytokines tumour necrosis factor-α (TNF-α) and interferon-γ (IFN-γ) and release cytotoxic molecules (perforin and granzyme B) that could harm the vascular wall by promoting inflammation and plaque rupture.^10^ We showed that co-stimulatory receptors OX40 and 4-1BB modulate CD28^null^ T-cell function and that co-stimulation blockade reduced the inflammatory and cytotoxic actions of these cells.^10^ We have also identified that CD28^null^ T cells from ACS patients have defects in proteasomal degradation of pro-apoptotic molecules.^5^^,^^11^^,^^12^ However, the precise mechanisms that regulate CD28^null^ T-cell expansion in ACS patients are yet to be deciphered.

Inflammatory cytokines such as TNF-α, interleukin-1β (IL-1β), and IL-6 have important roles in driving inflammation in CAD patients.^3^^,^^13–15^ Recently, the CANTOS trial showed that targeted cytokine inhibition (IL-1β inhibition with Canakinumab) in CAD patients with a prior myocardial infarction and residual inflammatory risk reduced further cardiovascular events.^16^ Other cytokines that are deregulated in chronic inflammatory diseases (e.g. rheumatoid arthritis, RA) and are currently targeted in patients are IL-7 and IL-15 that belong to the common gamma chain cytokine family.^17–19^ Whether these cytokines are involved in the expansion and function of CD28^null^ T cells in ACS patients is unknown.

Here, we provide novel evidence that the cytokines IL-7 and IL-15 trigger CD28^null^ T-cell expansion in ACS patients, while inflammatory cytokines (TNF-α, IL-1β, and IL-6) do not. IL-7- and IL-15-induced preferential activation and proliferation of CD28^null^ T cells and promoted their cytotoxic and inflammatory function. We dissect the mechanistic basis of IL-7 and IL-15 effects on CD28^null^ T cells from ACS patients and demonstrate that Tofacitinib, a selective JAK1/JAK3 inhibitor that blocks IL-7/IL-15 signalling, significantly inhibits CD28^null^ T-cell expansion. These new data suggest that targeting IL-7 and IL-15 could potentially provide a therapeutic strategy to prevent expansion of CD28^null^ T cells in ACS patients.

## 2. Methods

### 2.1 Study population

Peripheral blood was collected from patients with ACS (Supplementary material online, *Tables S1* and *S2*; samples collected <12 h from chest pain) admitted at the coronary care unit, St. George’s Hospital NHS Trust, London. Patients with malignancies, infectious diseases, autoimmune disorders, on treatment with anti-inflammatory drugs except aspirin, or over 80 years old were excluded from the study, as previously described.^10^^,^^11^ The study conformed to the Declaration of Helsinki principles (study approved by the London & Chelsea Research Ethics Committee, REC 09/H0801/27), and written informed consent was obtained from all study participants prior to inclusion in the study.

### 2.2 Flow cytometry

The frequency of CD28^null^ T cells in fresh peripheral blood samples (circulating CD28^null^ T cells) was determined by staining 100 µL blood with CD4-FITC and CD28-APC (BD Biosciences), followed by red blood cells lysis with Lyse/Fix buffer (BD Biosciences), as previously described.^10^ The percentage of circulating CD28^null^ T cells was calculated as the percentage of CD4^+^ T cells, as previously described.^10^ The gating strategy for quantification of CD28^null^ T cells in fresh peripheral blood samples and cultured peripheral blood mononuclear cells (PBMCs) or CD4^+^ T cells is depicted in Supplementary material online, *Figure S1*. Where indicated, CD127-PE (Invitrogen), CD122-PE, CD132-PE, CD215-PE (all BioLegend) were added to fresh peripheral blood samples stained with CD4-FITC and CD28-APC. The following monoclonal antibodies were used to identify CD28^null^ T cells in cultured samples and analyse the expression of functional markers on CD28^null^ and CD28^+^ T cells: CD4-FITC, CD28-APC, CD28-PE, HLA-DR-APC, CXCR3-PE, CCR7-PE, CD45RO-FITC, TNF-α-PE-Cy7 (all BD Biosciences/BD Pharmingen); CD14-PE (Miltenyi Biotec); CD69-APC, CD215-PE, CD45RA-PE-Cy7 (all Biolegend); CD62L-APC, Granzyme B-PE, IFN-γ-APC (all eBioscience); CCR5-PE, CD127-PE (all Invitrogen). Intracellular levels of IFN-γ and TNF-α were quantified in sorted CD4^+^ T cells cultured alone or with IL-7 or IL-15 for 3 days following 4 h stimulation of with phorbol myristate acetate, ionomycin, and brefeldin, as previously described.^6^^,^^7^^,^^10^ Dead cells were excluded from the analysis by staining with 7-aminoactinomycin D (7-AAD, BD Biosciences) or, for intracellular staining (i.e. granzyme-B, IFN-γ, and TNF-α), by labelling with ZombieYellow (Biolegend) or Fixable viability stain 575 V (BD Biosciences) prior to fixation and permeabilization using Cytofix/Cytoperm (BD Biosciences). Phosphorylated STAT5 was assessed via the BD Phosflow method using CD4-H7, CD28-APC, Alexa Fluor 488 anti-STAT5, Alexa Fluor 488 mouse IgG1 κ isotype control, BD Cytofix buffer, and BD Perm Buffer III (all BD Biosciences) as per the manufacturer’s instructions. Samples were acquired on a FACSCalibur (BD Biosciences) or Navios (Beckman Coulter) flow cytometer and data were analysed using FlowJo software v7.6 (FlowJo, LLC). Mean fluorescence intensity (MFI) was calculated by subtracting the MFI of samples stained with isotype control antibodies from the MFI of samples stained with antibodies against specific markers. The percentage increase in CD28^null^ T cells following cytokine treatment was calculated as: 100×(%CD28^null^ in treated samples−%CD28^null^ in untreated samples)/%CD28^null^ in untreated samples.

**Figure 1:**
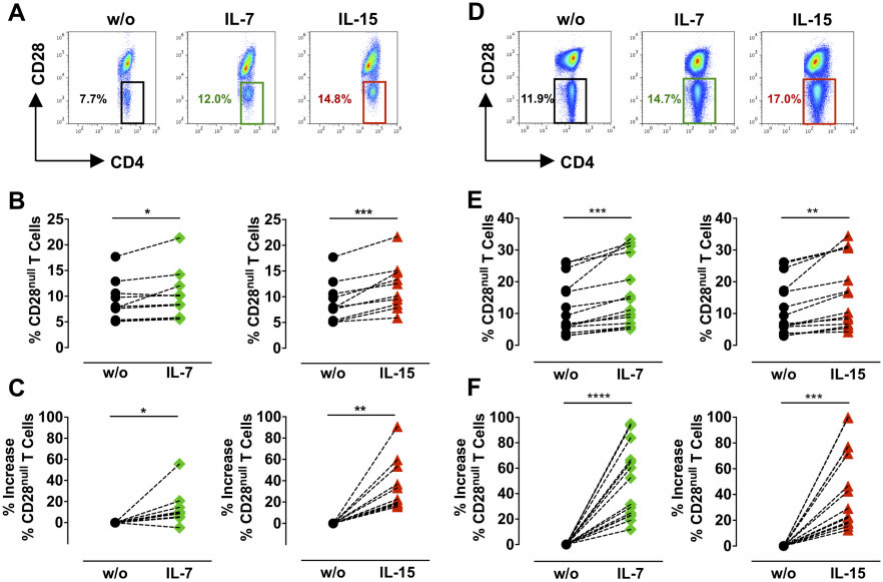
Effect of IL-7 and IL-15 on CD28^null^ T-cell expansion. PBMCs (*A–C n* = 10) or CD4^+^ T cells (*D–F n* = 14) from ACS patients with >3% circulating CD28^null^ T cells were treated with 50 ng/mL IL-7 or IL-15 for 4 days. (*A* and *D*) Illustrative dot plots show CD28^null^ T-cell percentage. (*B* and *E*) Graphs show CD28^null^ T-cell percentage, and (*C* and *F*) % increase in CD28^null^ T cells (calculated as described in Methods) in untreated (w/o) and cytokine-treated samples.*P <* *0.05; **P <* *0.01; ***P <* *0.001; ****P <* *0.0001 (paired two-tailed Student’s *t*-test).

### 2.3 Cell isolation and culture

Peripheral blood mononuclear cells were isolated from fresh blood samples by density gradient centrifugation as described previously.^11^ CD4^+^ T cells were purified by magnetic separation using negative selection kits from Miltenyi Biotec and Invitrogen (MagniSort) as per the manufacturers’ instructions. CD4^+^ T cells were cultured at 2×10^5^ cells per well in U-bottom 96-well plates in RPMI1640 (Life Technologies) containing 100 U/mL penicillin, 100 µg/mL streptomycin, and 15 mM l-glutamine (Sigma-Aldrich), supplemented with 5% pooled human AB serum (Corning). Cells were stimulated with the indicated concentrations of recombinant human cytokines (IL-1β, IL-6, IL-7, IL-15, TNF-α; R&D Systems) and cultured for up to 11 days. Where indicated, cells were stimulated with IL-7/IL-15 in the presence or absence of 100 nM Tofacitinib (CP 690550 citrate, Tocris Bioscience). Where indicated, culture supernatant from CD4^+^ T cells stimulated with IL-7, IL-15, and un-stimulated cells were collected and kept frozen until quantification of TNF-α and IFN-γ by DuoSet ELISA (R&D Systems), as per the manufacturer’s instructions.

### 2.4 Proliferation assay

Sorted CD4^+^ T cells were labelled with 1 µM carboxyfluorescein succinimidyl ester (CFSE, Invitrogen) and cultured in the presence of IL-7 or IL-15 as indicated. Proliferation (assessed as CFSE dilution) was quantified by flow cytometry following staining with antibodies (CD4-APC-H7, CD28-APC) and dead cell exclusion with 7-AAD (all BD Biosciences).

### 2.5 Degranulation assay

2×10^5^ CD4^+^ T cells were cultured alone or stimulated with 50 ng/mL IL-7 or IL-15 for 4 days. Four hours before analysis, CD107a-PE (BD Biosciences) was added to the culture and cells were either left un-stimulated or stimulated with 2 µg/mL functional grade anti-human CD3 monoclonal antibody (Invitrogen); BD GolgiStop (BD Biosciences) was added for the last 3 h of culture. Cells were then stained with CD4-FITC, CD28-APC, and 7-AAD (BD Biosciences) and CD107a expression was quantified by flow cytometry.

### 2.6 Quantification of plasma cytokine levels

Plasma was separated from fresh EDTA-treated blood samples from ACS patients and stored frozen until quantification of IL-7, IL-15, TNF-α, IL-1β, and IL-6 by DuoSet ELISA (R&D Systems) and by IL-7 and IL-15 Quantikine high sensitivity ELISA (R&D Systems).

### 2.7 Statistics

Statistical analysis was performed using GraphPad Prism v7.02 and v8.4. Cytokine-treated and untreated samples were compared using paired two-tailed Student’s *t*-test or two-tailed Wilcoxon matched-pairs signed-rank test as indicated. For comparison of more than two groups, statistical significance was determined using one- or two-way analysis of variance (ANOVA) and Bonferroni post-test for multiple comparisons. Plasma cytokine levels in the two study groups were compared using two-tailed Mann–Whitney test. Categorical data were analysed with the χ^2^ test. Probability (*P*) values of <0.05 were considered statistically significant.

### 2.8 Study approval

The study conformed to the Declaration of Helsinki principles (study approved by the London & Chelsea Research Ethics Committee, REC 09/H0801/27), and written informed consent was obtained from all study participants prior to inclusion in the study.

## 3. Results

### 3.1 Inflammatory cytokines TNF-α, IL-1β, and IL-6 do not induce expansion of CD28^null^ T cells from ACS patients

To investigate the role of inflammatory cytokines (TNF-α, IL-1β, IL-6) in CD28^null^ T lymphocyte expansion in ACS, we analysed cells from patients without expansion of the CD28^null^ T-cell subset defined as <2% circulating CD28^null^ T cells out of all CD4^+^ T cells in peripheral blood (quantified as detailed in Methods and Supplementary material online, *Figure S1A*).^6^^,^^7^^,^^10^ PBMCs from ACS patients were cultured alone or in the presence of cytokines for up to 7 days. The percentage of CD28^null^ T cells remained unchanged following TNF-α, IL-1β, or IL-6 treatment at all concentrations and time points tested (Supplementary material online, *Figure S2*). Similar results were observed with high-purity sorted CD4^+^ T cells treated with TNF-α, IL-1β, or IL-6, which did not affect CD28^null^ T-cell percentage (Supplementary material online, *Figure S3*). Combinations of inflammatory cytokines (i.e. TNF-α+IL-1β, TNF-α+IL-6, IL-1β+IL-6, and TNF-α+IL-1β+IL-6) had no effect on CD28^null^ T cells (Supplementary material online, *Figure S4*). To further investigate whether inflammatory cytokines have any effect on CD28^null^ T cells, we tested these cytokines on PBMCs (Supplementary material online, *Figure S5A*–*C*) or high-purity sorted CD4^+^ T cells (Supplementary material online, *Figure S5D*–*F*) from ACS patients with pre-existing CD28^null^ T-cell expansion in peripheral blood, that is, >3% circulating CD28^null^ T cells (detailed in Methods and Supplementary material online, *Figure S1A*).^6^^,^^7^^,^^10^ In all samples tested TNF-α, IL-1β, or IL-6 did not affect CD28^null^ T-cell percentage, indicating that these inflammatory cytokines are unlikely to contribute to CD28^null^ T-cell expansion in ACS patients.

**Figure 2:**
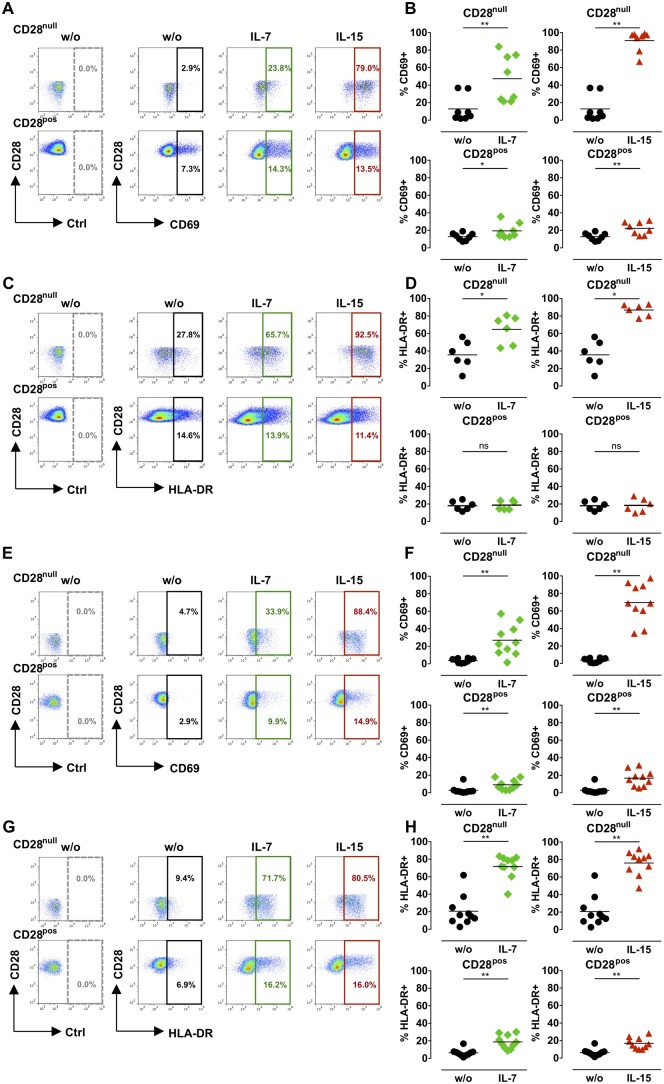
Effect of IL-7 and IL-15 on CD28^null^ T-cell activation. PBMCs (*A*–*D*) or CD4^+^ T cells (*E*–*H*) from ACS patients were treated with 50 ng/mL IL-7 or IL-15 for 4 days. The activation markers CD69 and HLA-DR were analysed on CD28^null^ and CD28^pos^ T cells. (*A* and *E*) Illustrative dot plots display CD69 expression on CD28^null^ and CD28^pos^ T cells; dashed gates, isotype control antibody (Ctrl). (*B* and *F*) Graphs show the percentage of CD69^+^ cells in untreated samples (w/o) or after cytokine treatment (*B n* = 8; *F n* = 10). (*C* and *G*) Illustrative dot plots display HLA-DR expression on CD28^null^ and CD28^pos^ T cells; dashed gates, isotype control antibody (Ctrl). (*D* and *H*) Graphs show the percentage of HLA-DR^+^ cells in untreated samples (w/o) or after cytokine treatment (*D n* = 6; *H n* = 10). *P <* *0.05;**P <* *0.01; ns, not significant (two-tailed Wilcoxon matched-pairs signed-rank test).

**Figure 3:**
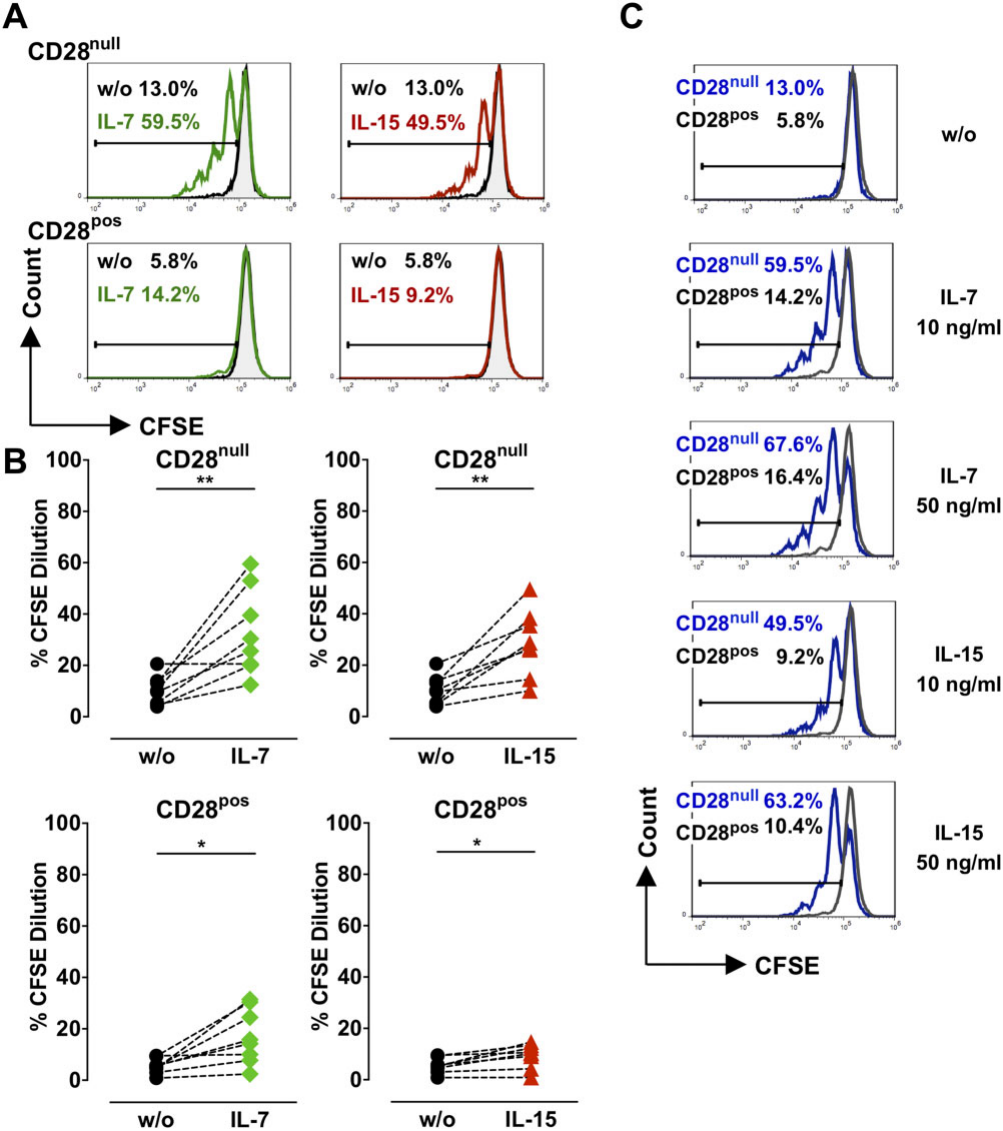
Effect of IL-7 and IL-15 on CD28^null^ T-cell proliferation. CD4^+^ T cells from ACS patients were labelled with CFSE and cultured in the presence of 10 or 50 ng/mL IL-7 or IL-15 for 5 days. (*A*) Histograms illustrate CFSE fluorescence in CD28^null^ and CD28^pos^ T cells following treatment with 10 ng/mL IL-7 or IL-15. The percentage of cells that have proliferated (diluted CFSE) is indicated above the linear gates. (*B*) Proliferation of CD28^null^ and CD28^pos^ T cells in untreated samples (w/o) and after cytokine treatment (*n* = 8). (*C*) Histograms show CFSE fluorescence of CD28^null^ (blue) and CD28^pos^ (black) T cells treated with the indicated cytokines. *P <* *0.05; **P <* *0.01 (paired two-tailed Student’s *t*-test).

**Figure 4:**
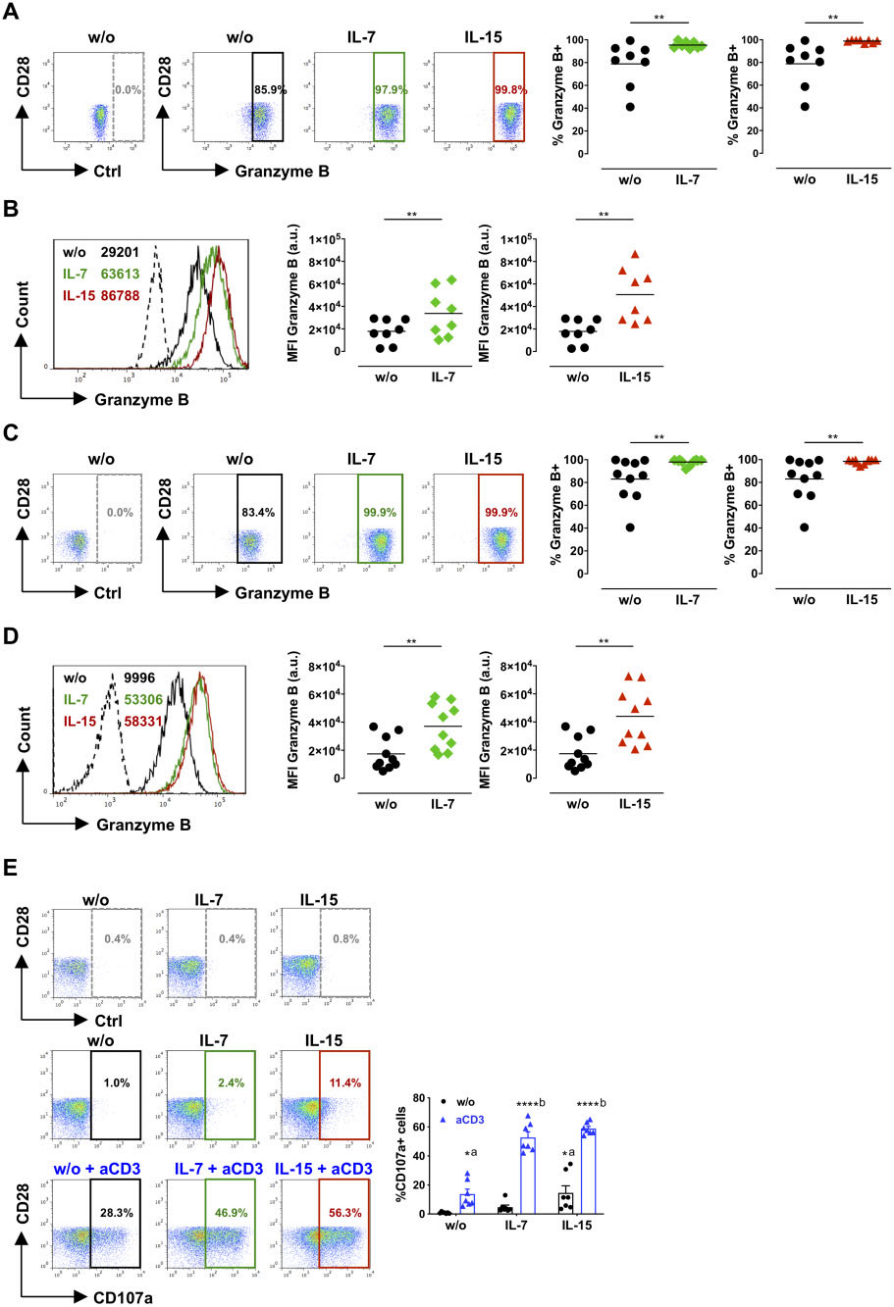
Effect of IL-7 or IL-15 on the production of granzyme B and degranulation of CD28^null^ T cells. PBMCs (*A* and *B n* = 8) or CD4^+^ T cells (*C* and *D n* = 10) from ACS patients were treated with 50 ng/mL IL-7 or IL-15 for 4 days. (*A* and *C*) Illustrative dot plots show Granzyme B (GzB) expression in CD28^null^ T cells; dashed gates, isotype control antibody (Ctrl). The graphs depict percentages of GzB^+^ cells in untreated samples (w/o) and after cytokine treatment. (*B* and *D*) Histograms showing GzB expression in CD28^null^ T cells; dashed histograms, isotype control antibody. The numbers indicate the mean fluorescence intensities (MFI) in cells cultured alone (w/o) or with cytokines. The graphs show GzB levels (MFI) in untreated and cytokine-treated cells. **P <* *0.01 (two-tailed Wilcoxon matched-pairs signed-rank test). a.u., arbitrary units. (*E*) CD4^+^ T cells (*n* = 7) were cultured alone (w/o) or with 50 ng/mL IL-7 or IL-15 for 4 days. On day 4, cells were stimulated with anti-CD3 antibodies (aCD3) for 4 h as indicated, and degranulation was quantified with CD107a (detailed in Methods). Illustrative dot plots show CD107a expression in CD28^null^ T cells; dashed gates, isotype control antibody (Ctrl); the graph displays the percentage of CD28^null^ T cells expressing CD107a (mean±SEM). *^a^*P *<* *0.05 (aCD3 only or IL-15 only vs. w/o); ****^b^*P *<* *0.0001 (aCD3+IL-7 or aCD3+IL-15 vs. aCD3) (two-way ANOVA with post-test Tukey for multiple comparisons).

**Figure 5:**
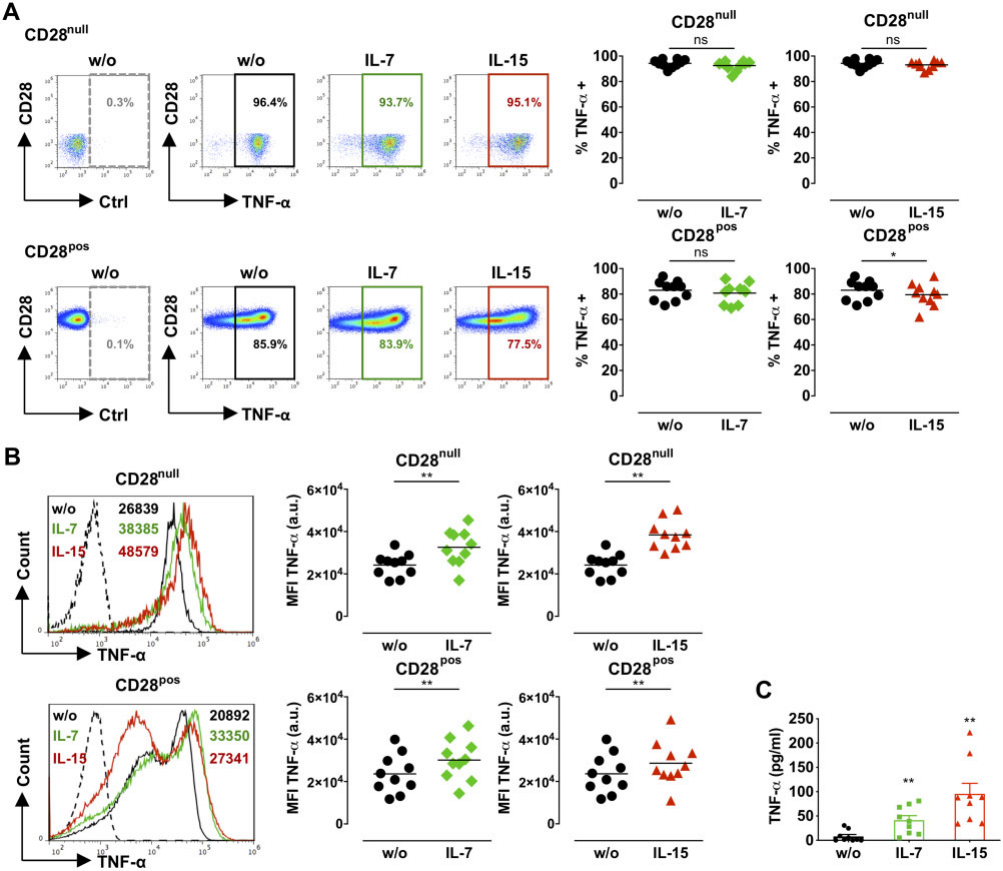
Effect of IL-7 or IL-15 on the production of TNF-α by CD28^null^ T cells. CD4^+^ T cells (*n* = 10) from ACS patients were treated with 50 ng/mL IL-7 or IL-15 for 3 days. (*A*) Illustrative dot plots and graphs display the percentage of TNF-α^+^CD28^null^ (top) and TNF-α^+^CD28^pos^ (bottom) T cells in untreated samples (w/o) and after cytokine treatment; dashed gates, isotype control antibody (Ctrl). (*B*) The histograms and graphs show TNF-α expression levels by CD28^null^ (top) and CD28^pos^ (bottom) T cells with or without (w/o) cytokine treatment; dashed histograms, isotype control antibody; the numbers indicate the mean fluorescence intensities (MFI) for each treatment. (*A* and *B*) *P <* *0.05; **P <* *0.01; ns, not significant (two-tailed Wilcoxon matched-pairs signed-rank test). a.u., arbitrary units. (*C*) The bar graph shows TNF-α levels in media from CD4^+^ T cells (*n* = 9) cultured with cytokines as above (mean±SEM). **P <* *0.01 (one-way ANOVA with post-test Bonferroni for multiple comparisons).

### 3.2 IL-7 and IL-15 trigger expansion of CD28^null^ T cells from ACS patients

As inflammatory cytokines did not affect the number of CD28^null^ T cells, we sought whether other cytokines drive expansion of this subset. We focused on IL-7 and IL-15, which belong to the common gamma chain cytokine family as they are involved in the pathogenesis of chronic inflammatory disorders and are targeted in patients with autoimmune disorders.^17^ PBMCs or high-purity sorted CD4^+^ T cells from ACS patients with >3% circulating CD28^null^ T cells were treated with IL-7 and IL-15. CD28^null^ T-cell percentage increased significantly on days 4 and 7 of culture (PBMC: *Figure 1A–C*, Supplementary material online, *Figure S6A*–*D*; CD4^+^ T cells: *Figure 1D*–*F*, Supplementary material online, *Figure S6E* and *F*). Combinations of IL-7 and IL-15 did not have synergistic effects on CD28^null^ T-cell expansion (not shown).

**Figure 6:**
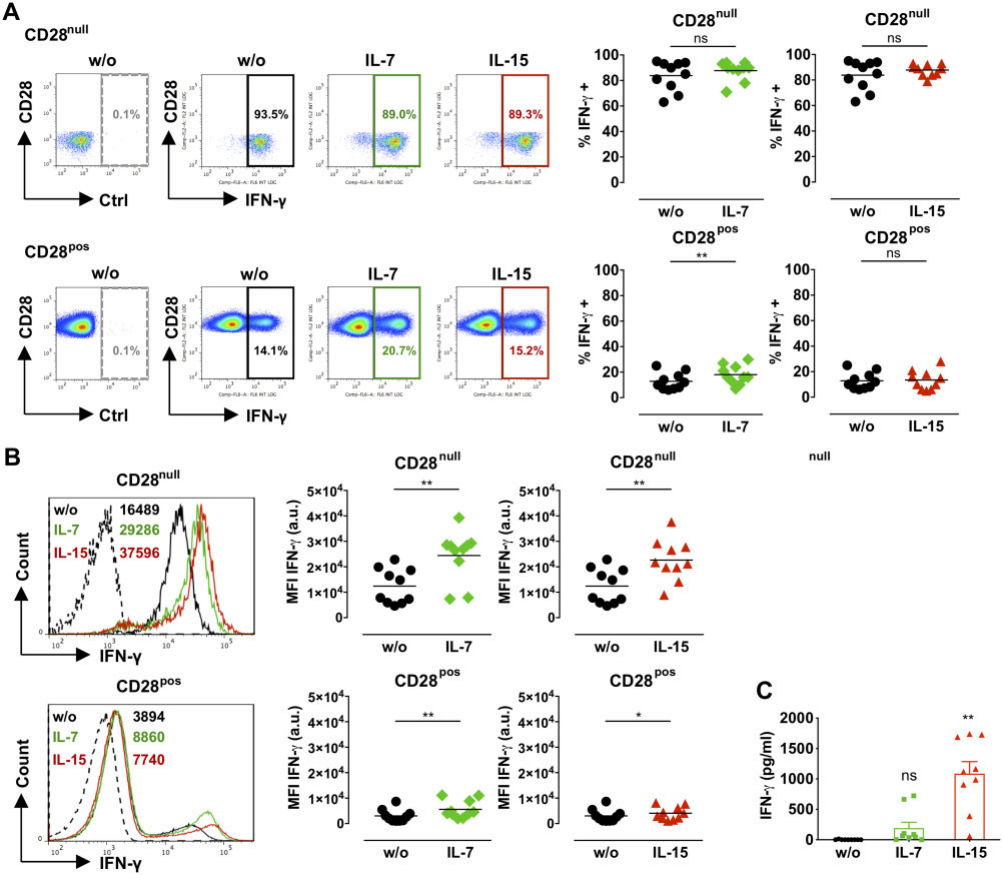
Effect of IL-7 or IL-15 on the production of IFN-γ by CD28^null^ T cells. CD4^+^ T cells from ACS patients (*n* = 10) were treated with 50 ng/mL IL-7 or IL-15 for 3 days. (*A*) Illustrative dot plots and graphs display the percentage of IFN-γ^+^CD28^null^ (top) and IFN-γ^+^CD28^pos^ (bottom) T cells in untreated samples (w/o) and after cytokine treatment; dashed gates, isotype control antibody (Ctrl). (*B*) The histograms and graphs show IFN-γ expression levels by CD28^null^ (top) and CD28^pos^ (bottom) T cells with or without (w/o) cytokine treatment; dashed histograms, isotype control antibody; the numbers indicate the mean fluorescence intensities (MFI) for each treatment. (*A* and *B*) *P <* *0.05; **P <* *0.01; ns, not significant (two-tailed Wilcoxon matched-pairs signed-rank test). a.u., arbitrary units. (*C*) The bar graph shows IFN-γ levels in media from CD4^+^ T cells (*n* = 9) cultured with cytokines as above (mean±SEM). **P <* *0.01; ns, not significant (one-way ANOVA with post-test Bonferroni for multiple comparisons).

We also examined whether IL-7 and IL-15 expand CD28^null^ T cells from ACS patients with <2% CD28^null^ T cells in peripheral blood. In PBMCs, IL-15 significantly increased CD28^null^ T-cell percentage at days 4 and 7 of culture (Supplementary material online, *Figure S7*), while in sorted CD4^+^ T cells both IL-7 and IL-15 increased CD28^null^ T-cell percentage at days 7 and 11 (Supplementary material online, *Figure S8*).

**Figure 7:**
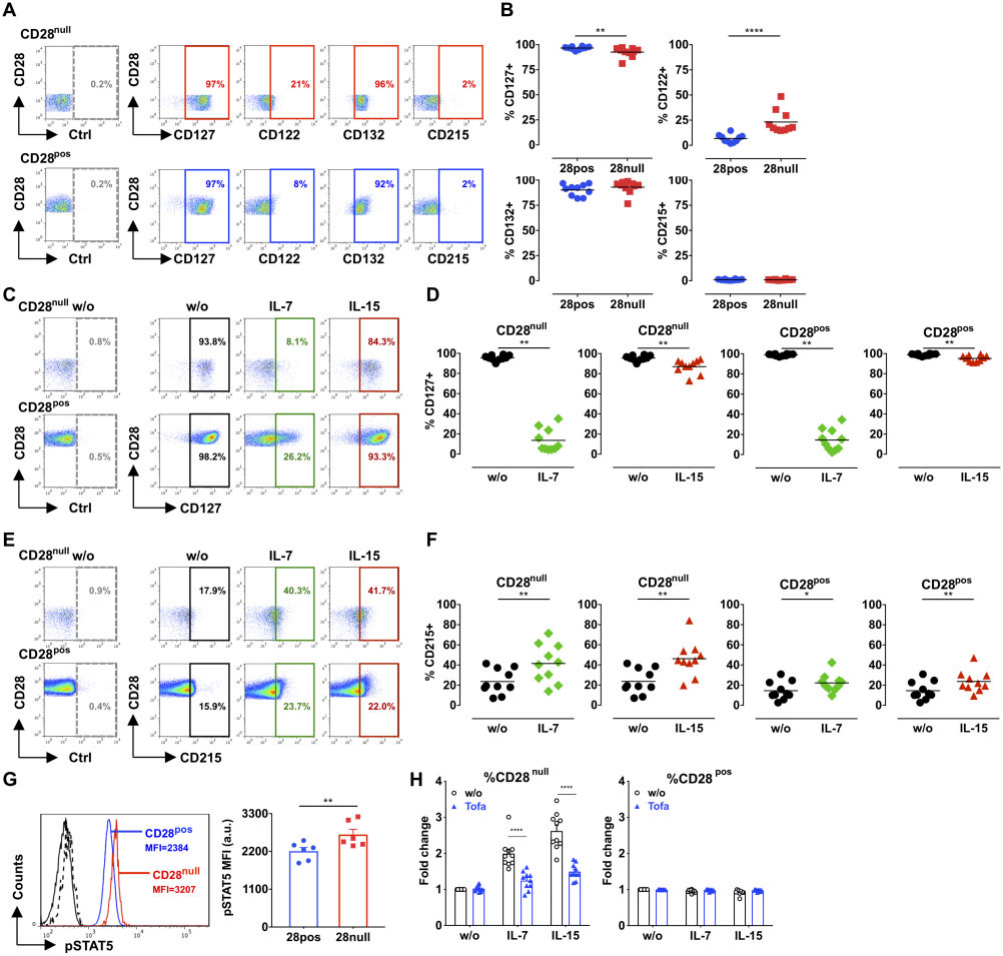
Blockade of IL-7/IL-15 signalling with the JAK1/JAK3 selective inhibitor Tofacitinib prevents CD28^null^ T-cell expansion. Fresh peripheral blood samples from ACS patients (*n* = 10) were stained with CD4, CD28, CD122, CD127, CD132, and CD215 monoclonal antibodies. Illustrative dot plots (*A*) and graphs (*B*) show the percentage of CD28^null^ and CD28^+^ T cells expressing CD122, CD132, CD127, and CD215; dashed gates, isotype control antibody (Ctrl). CD4^+^ T cells from ACS patients (*n* = 10) were cultured alone (w/o) or treated with 50 ng/mL IL-7 or IL-15 for 3–4 days and expression of CD127 and CD215 was analysed on CD28^null^ and CD28^pos^ T cells. Illustrative dot plots and graphs display the percentage of CD127^+^ cells (*C* and *D*), and CD215^+^ cells (*E* and *F*) in CD28^null^ and CD28^pos^ T cells; dashed gates, isotype control antibody (Ctrl). (*G*) Baseline levels of phosphorylated STAT5 (pSTAT5) were quantified using the PhosFlow Method (described in Methods); black and dashed histograms, isotype control antibody; the numbers indicate the mean fluorescence intensity (MFI). Illustrative histograms and bar graph display the expression levels (MFI) of pSTAT5 in CD28^null^ and CD28^pos^ T cells (*n* = 6; mean±SEM). (*H*) CD4^+^ T cells from ACS patients (*n* = 10) were cultured alone (w/o) or treated with 50 ng/mL IL-7 or IL-15 in the presence or absence of 100 nM Tofacitinib (Tofa) for 7 days. Graphs show the fold change in the percentage of CD28^null^ or CD28^pos^ T cells (mean±SEM). *P <* *0.05; **P <* *0.01; ***P <* *0.001; ****P <* *0.0001. (*B*) Two-tailed Mann–Whitney test; (*D* and *F*) two-tailed Wilcoxon matched-pairs signed-rank test; (*G*) paired two-tailed Student’s *t*-test; (*H*) two-way ANOVA with post-test Bonferroni for multiple comparisons.

### 3.3 IL-7 and IL-15 activate CD28^null^ T cells from ACS patients

Next, we sought the underlying mechanisms through which IL-7 and IL-15 induced CD28^null^ T-cell expansion. T-cell activation is a pre-requisite for lymphocyte expansion and therefore, we quantified the activation markers CD69 and HLA-DR following cytokine treatment. In both PBMCs (*Figure 2A–D*) and sorted CD4^+^ T cells (*Figure 2E–H*), IL-7 and IL-15 significantly increased the percentage of CD69-expressing and HLA-DR-expressing CD28^null^ T cells. In contrast, less marked changes were observed in the control CD28^+^ T-cell subset following IL-7 or IL-15 treatment (*Figure 2*). Inflammatory cytokines TNF-α, IL-1β, and IL-6 had no effect on CD69 or HLA-DR (Supplementary material online, *Figure S9*), in line with the failure of these cytokines to induce CD28^null^ T-cell expansion.

We also investigated the effects of IL-7 and IL-15 on chemokine receptors (CCR5, CXCR3) and memory markers (CD62L, CCR7, CD45RA, CD45RO). IL-7 and IL-15 significantly up-regulated CCR5 expression on CD28^null^ but not CD28^+^ T cells (Supplementary material online, *Figure S10A* and *B*), while CXCR3 (Supplementary material online, *Figure S10C* and *D*) and memory markers (Supplementary material online, *Figure S11*) were unchanged.

### 3.4 IL-7 and IL-15 induce proliferation of CD28^null^ T cells from ACS patients

As IL-7 and IL-15 preferentially activate CD28^null^ T cells (*Figure 2*), we investigated whether this resulted in proliferation of this cell subset, which may explain CD28^null^ T-cell expansion in ACS. For this purpose, sorted CD4^+^ T cells were labelled with CFSE and cultured with IL-7 or IL-15; proliferation, assessed as CFSE-dilution, was quantified (detailed in Methods). There was little basal proliferation of CD28^null^ and control CD28^+^ T cells in the absence of IL-7 and IL-15 on days 5 and 7 of culture (*Figure 3*, Supplementary material online, *Figure S12*). IL-7 and IL-15 induced significant proliferation of CD28^null^ T cells, while less proliferation was noted in CD28^+^ T cells (*Figure 3*, Supplementary material online, *Figure S12*).

### 3.5 IL-7 and IL-15 increase granzyme B production and degranulation of CD28^null^ T cells from ACS patients

Next, we tested whether IL-7 and IL-15 affect CD28^null^ T-cell function. These cells characteristically produce the cytotoxic molecule GzB, which endows them with cell lytic function, in stark contrast to conventional CD28^+^ T cells that do not express this molecule.^10^ PBMCs (*Figure 4A* and *B*) or sorted CD4^+^ T cells (*Figure 4C* and *D*) from ACS patients were treated with IL-7 or IL-15 for 4 days. In line with previous studies,^10^ most CD28^null^ T cells (79% for PBMCs *Figure 4A*; 83% for CD4^+^ T cells *Figure 4C*) expressed GzB at baseline. Both IL-7 and IL-15 significantly increased the percentage of CD28^null^ T cells expressing GzB (*Figure 4A* and *C*). Moreover, IL-7 and IL-15 also increased the expression levels (MFI) of GzB in CD28^null^ T cells (*Figure 4B* and *D*). Next, we investigated whether IL-7 and IL-15 affect the cytotoxic function of CD28^null^ T cells using a degranulation assay based on CD107a expression, as previously described.^10^ Degranulation is triggered by T-cell receptor (TCR) stimulation either by antigen recognition or, in *in vitro* assays by anti-CD3 antibodies, and degranulating cells are identified by the expression of CD107a (lysosomal associated membrane protein-1) on the cell surface.^20^ Both IL-7 and IL-15 significantly up-regulated the anti-CD3-induced degranulation of CD28^null^ T cells (*Figure 4E*). Notably, IL-15 but not IL-7 induced CD28^null^ T-cell degranulation even in the absence of anti-CD3 stimulation (*Figure 4E*).

### 3.6 IL-7 and IL-15 augment TNF-α and IFN-γ production by CD28^null^ T cells from ACS patients

CD28^null^ T cells from ACS patients produce high levels of TNF-α and IFN-γ.^10^ As described previously,^10^ the percentage of TNF-α (*Figure 5A*) and IFN-γ (*Figure 6A*) producing cells was higher in resting CD28^null^ (TNF-α 94.3%; IFN-γ 83.8%) than in the control CD28^+^ T-cell subset (TNF-α 83%; IFN-γ 12.8%). IL-7 or IL-15 did not change the percentage of TNF-α-producing CD28^null^ T cells (*Figure 5A*), however, TNF-α expression levels (MFI) significantly increased in both CD28^null^ and CD28^+^ T cells (*Figure 5B*). This was accompanied by a significant increase in TNF-α in the culture medium of CD4^+^ T cells treated with IL-7 or IL-15 (*Figure 5C*). While IL-7 or IL-15 did not change the percentage of IFN-γ-producing CD28^null^ T cells (*Figure 6A*), they markedly up-regulated IFN-γ expression levels (MFI) in CD28^null^ T cells (*Figure 6B*), while this was less prominent in control CD28^+^ T cells (*Figure 6B*). IFN-γ levels in culture medium from cytokine-treated CD4^+^ T cells were significant increased by IL-15 (*Figure 6C*).

### 3.7 Blocking IL-7 and IL-15 signalling prevents the expansion of CD28^null^ T cells from ACS patients

We next quantified IL-7 and IL-15 in plasma from ACS patients with CD28^null^ T-cell expansion (>3% CD28^null^ T cells in peripheral blood) and those without expansion of this cell subset (<2% CD28^null^ T cells). IL-7 and IL-15 levels were very low in plasma (Supplementary material online, *Figure S13A*), in keeping with other studies that showed that these cytokines are mainly expressed within cells and are barely detectable in circulation.^21^^,^^22^ Mean IL-7 and IL-15 levels were higher in ACS patients with CD28^null^ T-cell expansion (IL-7: 8.08 pg/mL; IL-15: 11.01 pg/mL) compared to ACS patients without expansion of this cell subset (IL-7: 3.36 pg/mL; IL-15: 3.53 pg/mL), but the differences were not significant (Supplementary material online, *Figure S13A*). TNF-α, IL-1β, and IL-6 plasma levels were also low and similar in the two ACS groups (Supplementary material online, *Figure S13B*). We hypothesized that differences in signalling downstream of IL-7/IL-15 receptors may render CD28^null^ T cells more sensitive to these cytokines. The IL-7 receptor (IL-7R) has two chains: CD127 (the α chain, found only in IL-7R); and CD132 (the γ chain) that is shared with the IL-15 receptor.^23^ The IL-15 receptor (IL-15R) is composed of CD215 (unique to IL-15R; binds IL-15 but is not required for IL-15 signalling); CD122 (the β chain), which is shared with the IL-2 receptor; and CD132.^24^ We assessed the baseline expression of these chains in CD28^null^ and in the control CD28^+^ T-cell subset directly in fresh peripheral blood samples from ACS patients. CD127 expression was lower in CD28^null^ T cells compared to CD28^+^ T cells (*Figure 7A* and *B*; *P* < 0.01). CD215 levels were very low in both cell subsets (*Figure 7A* and *B*), in keeping with previous studies suggesting that CD215 is not expressed in resting T cells but mainly expressed on cells (e.g. dendritic cells) that produce and present IL-15 to responding T cells.^25^ Notably, CD28^null^ T cells expressed significantly higher levels of CD122 compared to control CD28^+^ T cells (*Figure 7A* and *B*; *P* < 0.0001), which may explain their increased responsiveness to IL-15. CD132 expression was comparable in the two cell subsets (*Figure 7A* and *B*). Next, we assessed the effect of IL-7 and IL-15 on the expression of CD127 and CD215 (the chains involved in binding IL-7 and IL-15, respectively) in CD28^null^ and CD28^+^ T cells. IL-7 markedly down-regulated CD127 in both CD28^null^ and CD28^+^ T cells (*Figure 7C* and *D*). Interestingly, IL-15 also significantly down-regulated CD127 but to a lower extent compared to IL-7 (*Figure 7C* and *D*). In contrast, CD215 was significantly up-regulated in response to IL-7 and IL-15 mainly in CD28^null^ T cells (*Figure 7E* and *F*). IL-7 and IL-15 receptors signal preferentially through JAK1/JAK3/STAT5, which associate with CD127, CD122, and CD132.^17^^,^^23^^,^^24^^,^^26^ Baseline levels of phosphorylated-STAT5 were significantly higher in un-stimulated CD28^null^ T cells from ACS patients, compared to the control CD28^+^ T-cell subset (*Figure 7G*), indicating higher basal activation of IL-7/IL-15 signalling in CD28^null^ T cells. Next, we blocked IL-7/IL-15 signalling with the JAK1/JAK3 selective inhibitor Tofacitinib,^27^^,^^28^ which significantly inhibited CD28^null^ T-cell expansion, while not affecting CD28^+^ T cells (*Figure 7H*).

## 4. Discussion

Here, we show that IL-7 and IL-15 cytokines augment the number and function of CD28^null^ T cells from patients with ACS. We demonstrate that IL-7 and IL-15 trigger expansion of CD28^null^ T cells, while inflammatory cytokines TNF-α, IL-1β, and IL-6 have no effect. The mechanisms underlying CD28^null^ T-cell expansion are preferential activation and proliferation of CD28^null^ T cells by IL-7 and IL-15. These cytokines increase the cytotoxic function of CD28^null^ T cells (GzB production and degranulation) and the production of IFN-γ. We provide further mechanistic insights showing marked differences in baseline levels of CD127 and CD122 (component chains of IL-7 and IL-15 receptors) and increased baseline phosphorylation of STAT5 in CD28^null^ T cells compared to their control CD28^+^ T-cell subset. Blockade of IL-7/IL-15 signalling with a JAK1/JAK3 selective inhibitor prevents the expansion of CD28^null^ T cells from ACS patients (summarized in the *Graphical Abstract*).

Inflammatory CD28^null^ T cells expand in the circulation and atherosclerotic plaques of patients with ACS.^6^^,^^7^ Notably, ACS patients with expansion of the CD28^null^ T-cell subset have increased risk for recurrent severe acute coronary events and poorer prognosis compared to ACS patients without CD28^null^ T-cell expansion.^9^ This study also showed that an increase in CD28^null^ T-cell frequency is an independent predictor of future acute coronary events in ACS patients.^9^ The precise mechanisms driving the expansion of CD28^null^ T cells in ACS are unknown. Here, we show that the cytokines IL-7 and IL-15 are involved in the expansion of CD28^null^ T cells from ACS patients, while inflammatory cytokines TNF-α, IL-1β, and IL-6 did not have an effect. However, inflammatory cytokines TNF-α, IL-1β, and IL-6 are important in atherosclerosis pathogenesis, as recently shown by the CANTOS trial, which found that specifically targeting inflammation (IL-1β inhibition) improves clinical outcomes in patients with a previous myocardial infarction.^16^ Our findings underscore that several cytokines and inflammatory networks may be at work in ACS patients, and that a better characterization of patients’ inflammatory status is needed to improve the efficacy of anti-inflammatory therapies in ACS. Moreover, as CD28^null^ T cells have been exclusively identified in humans and do not exist in mice, this precludes interrogation of their roles in atherosclerosis using currently available animal models. Our work emphasizes the need to investigate inflammation and immune responses in patients with ACS as key information on the cellular and molecular pathophysiology of human CAD may be missed in murine models.

Strikingly, IL-7 and IL-15 drove the activation and proliferation of CD28^null^ T cells from ACS patients in the absence of T-cell receptor (TCR)-derived signals (i.e. antigen stimulation). A possible explanation is that CD28^null^ T cells share features with natural killer (NK) cells^29^^,^^30^ that also expand in response to IL-15 in the absence of antigen-dependent stimulation. IL-15 has central roles in the development, maintenance, activation, and effector function of NK cells (cytotoxicity, IFN-γ production).^31^ Indeed, CD28^null^ T cells express NK cell receptors and exhibit NK-like effector functions such as expression of cytotoxic molecules (GzB, perforin), cytotoxicity, and IFN-γ production,^29^^,^^30^ which may explain their response to IL-15. In contrast to IL-15, IL-7 does not increase NK cell activation, cytotoxicity, and IFN-γ production.^32^ Nevertheless, IL-7 triggered activation and markedly augmented the cytotoxic and cytokine production function of CD28^null^ T cells from ACS patients. Moreover, IL-7 had comparable effects to IL-15 on CD28^null^ T-cell expansion, activation, and function, although only IL-15 directly triggered CD28^null^ T-cell degranulation in the absence of anti-CD3 stimulation. Notably, our data that IL-7 and IL-15 drive CD28^null^ T-cell activation, proliferation, and function independently of TCR signals indicate that these cytokines may sustain the expansion and functions of these lymphocytes in the absence of antigen restimulation in ACS. IL-7 and IL-15 also increased CCR5 expression on CD28^null^ T cells from ACS patients. CCR5 has been suggested to regulate tissue homing to the aorta of Th1 CD4^+^ T cells.^33^ This suggests that IL-7 and IL-15 may enhance CD28^null^ T-cell homing to atherosclerotic plaques, which will be investigated in future studies.

IL-7 and IL-15 have been implicated in several autoimmune disorders, in particular in RA where these cytokines are identified in synovial fluid and tissue.^18^^,^^19^ The effect of IL-7 on CD28^null^ T cells has not been previously studied in any disease, while the effect of IL-15 on CD28^null^ T cells has been investigated in multiple sclerosis, elderly individuals, and rheumatoid arthritis.^34–36^ Whether IL-7 and IL-15 have a role in atherosclerosis is less well established. Expression of IL-15 in lipid-rich human atherosclerotic plaques associated with increased T-cell infiltration and plaque-derived T-cell lines proliferated to IL-15 *in vitro*.^35^ In addition, IL-15 induced the development of atherosclerotic plaques in *ldlr*^−/−^ mice, though this is independent of CD28^null^ T cells as this cell subset is not described in mice.^37^ While a previous study suggested that plasma IL-7 is raised in unstable or stable angina patients compared to healthy subjects,^38^ others reported that IL-7 and IL-15 are predominantly expressed within cells (macrophages, dendritic cells, epithelial, and stromal cells) with barely detectable circulating stores.^21^^,^^22^ We found low plasma levels of IL-7 and IL-15 in ACS patients, suggesting that CD28^null^ T-cell expansion in ACS may be driven by IL-7 and IL-15 produced locally in atherosclerotic plaques or artery-associated lymphoid tissue. Our data that baseline expression of CD127 was lower in CD28^null^ T cells from ACS patients compared to control CD28^+^ T cells, suggest down-regulation of CD127 by *in vivo* exposure to IL-7/IL-15. This is supported by our *in vitro* findings that CD127 is down-regulated in CD28^null^ T cells following IL-7 and IL-15 treatment. Additionally, the higher basal levels of phosphorylated-STAT5 in CD28^null^ T cells indicate higher basal activation of IL-7/IL-15 signalling *in vivo*. The increased responsiveness of CD28^null^ T cells to IL-15 is also supported by our new data that CD28^null^ T cells from ACS patients have higher baseline expression of CD122, the β-chain of IL-15 receptor. Moreover, IL-7 and IL-15 markedly up-regulated the expression of CD215 (the IL-15 receptor-specific chain) in CD28^null^ T cells *in vitro*, which would further augment their responsiveness to IL-15.

ACS patients with CD28^null^ T-cell expansion have increased risk for recurrent ACS and poor prognosis^9^ and may benefit from strategies that prevent expansion of these cells such as IL-7/IL-15 neutralization or signalling blockade. Indeed, we include novel data that Tofacitinib (a selective JAK1/JAK3 inhibitor that blocks IL-7/IL-15 signalling) prevents the expansion of CD28^null^ T cells from ACS patients. This effect of Tofacitinib on CD28^null^ T cells has not been explored in other diseases. Of interest, Tofacitinib is used in patients with RA and active psoriatic arthritis^39^^,^^40^ and has been recently approved for use in ulcerative colitis.^41^

A potential limitation of this study is that CD28^null^ T cells have been exclusively identified in humans and do not exist in mice precluding interrogation of their roles in atherosclerosis using currently available animal models. Therefore our study is an in-depth *ex vivo* analysis of fresh peripheral blood and primary cells from ACS patients. However, our work also highlights the need to investigate inflammation and immune responses in patients with ACS as key mechanisms of the cellular pathophysiology of human CAD may be missed in murine models.

## Conclusions

This study provides new mechanistic insights and identifies novel roles for IL-7 and IL-15 cytokines in CD28^null^ T-cell expansion and function in ACS. Our new findings suggest potential clinical applications of IL-7/IL-15 blockade in ACS patients with CD28^null^ T-cell expansion for targeted modulation of the inflammation mediated by these cells.

## Data availability

The data underlying this article are available in the article and in its Supplementary material online.

## Supplementary material

Supplementary material is available at *Cardiovascular Research* online.

## Authors’ contributions

Research studies design: I.E.D. and J.B. Conducting experiments, data acquisition, and analysis: J.B., V.M., S.K., I.C., P.D., and I.E.D. Original draft writing, reviewing, and editing: J.B. and I.E.D. Funding acquisition and administration: I.E.D. Provision of resources: Z.M.J.A. and J.C.K. Supervision: I.E.D.

## Supplementary Material

cvaa202_Supplementary_Data

## Notes

Time for primary review: 64 days

## Notes

Translational perspective CD28^null^ T cells expansion in ACS patients is an independent predictor of future acute coronary events and poor prognosis. The precise mechanisms underlying CD28^null^ T-cell expansion in ACS remain elusive. We show that IL-7 and IL-15 cytokines cause expansion of CD28^null^ T cells from ACS patients by triggering activation and proliferation, and augment the cytotoxic function of these cells and production of inflammatory cytokines. We demonstrate that CD28^null^ T-cell expansion is inhibited by Tofacitinib that specifically blocks IL-7/IL-15 signalling. Further dissection of the roles of IL-7/IL-15 may lead to more effective and specific anti-inflammatory therapies in ACS.
