# Supplementary material for: Interleukin-7 and interleukin-15 drive CD4+CD28null T lymphocyte expansion and function in patients with acute coronary syndrome
Source: Cardiovasc Res. 2020 Jul 9;117(8):1935–48. doi: 10.1093/cvr/cvaa202 (PMC8262639; doi:10.1093/cvr/cvaa202)

Supplementary Data

Interleukin-7 and interleukin-15 drive $CD4^{+}CD28^{null}$ T lymphocyte expansion and function in patients with acute coronary syndrome

Short title: IL-7 & IL-15 drive $CD28^{null}$ T-cell expansion and function

Jessica Bullenkamp PhD[1,2], Veronica Mengoni BA Nursing[1,2], Satdip Kaur PhD[1,2], Ismita Chhetri BSc[1,2], Paraskevi Dimou BSc[1,2], Zoë MJ Astroulakis MBBS BSc, PhD, FRCP[2], Juan Carlos Kaski DSc, MD, DM (Hons), FRSM, FRCP, FESC, FACC, FAHA[1,2] and Ingrid E. Dumitriu MD, PhD, FESC[1,2,3*]

[1]Molecular and Clinical Sciences Research Institute, St. George's, University of London, London, UK; [2]Cardiology Clinical Academic Group, St George's University Hospitals NHS Foundation Trust, London, UK; [3]Institute of Cardiovascular Sciences, University of Birmingham, Birmingham, UK (current affiliation)

Supplementary Data Inventory:

1. Supplemental Tables 1 and 2
2. Supplemental Figure legends
3. Supplemental Figures 1-13

Supplemental Table 1. ACS patients' characteristics (data included in Figures 1-7 and Supplemental Figures 1-12)

	**ACS patients**
Number (n)	83
Age, years (mean±SD)	64±13
Gender % (male/female)	77/23
Ethnicity % (C/A/B/O)*	64/24/4/8
Body mass index (mean±SD)	26.74±5.63
Family history of CAD (%)	36
Diabetes (type 2) (%)	24
Hypertension (%)	48
Current smoking (%)	33
Hypercholesterolemia (%)	36
Prior MI† (%)	14
Prior CABG‡ (%)	4
Prior PCI§ (%)	8
Type of MI % (STEMI/NSTEMI)||	90/10
Cholesterol (mmol/L) (mean±SD)	4.85±1.14
LDL# (mmol/L) (mean±SD)	2.81±1.00
HDL** (mmol/L) (mean±SD)	1.35±0.61
Triglycerides	1.54±1.70
Aspirin (prior to admission, %)	20
Statin (prior to admission, %)	23

*C/A/B/O, Caucasian/Asian/Black/Other; †MI, myocardial infarction; ‡CABG, coronary artery bypass grafting; §PCI, percutaneous coronary intervention; ||STEMI, ST-segment elevation MI; #LDL, low-density lipoprotein; **HDL, high-density lipoprotein

Supplemental Table 2. ACS patients' characteristics (data included in Supplemental Figure 13)

	ACS>3% CD28^{null}	ACS<2% CD28^{null}
Number (n)	38	38
Age, years (mean±SD)	65±13	60±13
Gender % (male/female)	71/29	79/21
Ethnicity % (C/A/B)*	58/40/2	82/16/2
Body mass index (BMI, mean±SD)	25.24±4.01	27.42±4.42
Family history of CAD (%)	50	45
Diabetes (type 2) (%)	26	29
Hypertension (%)	42	55
Current smoking (%)	34	42
Hypercholesterolemia (%)	24	26
Prior MI† (%)	11	16
Prior CABG‡ (%)	5	8
Prior PCI§ (%)	5	10
Type of MI % (STEMI/NSTEMI)||	95/5	97/3
Cholesterol (mmol/L) (mean±SD)	4.71±1.02	4.67±0.93
LDL# (mmol/L) (mean±SD)	2.79±0.88	2.95±0.91
HDL** (mmol/L) (mean±SD)	1.32±0.37	1.10±0.23
Triglycerides	1.28±0.88	1.53±1.23
Aspirin (prior to admission, %)	16	13
Statin (prior to admission, %)	18	23

*C/A/B, Caucasian/Asian/Black; †MI, myocardial infarction; ‡CABG, coronary artery bypass grafting; §PCI, percutaneous coronary intervention; ||STEMI, ST-segment elevation MI; #LDL, low-density lipoprotein; **HDL, high-density lipoprotein; BMI ($p<0.05$); no other significant differences were identified between the two groups

Supplemental Figures Legends

Supplemental Figure 1. Illustration of the gating strategy for quantification of CD28^{null} T cells in fresh peripheral blood samples and cultured cells from acute coronary syndrome (ACS) patients. (A) Circulating CD4^{+}CD28^{null} (CD28^{null}) T cells were quantified in fresh peripheral blood samples from ACS patients by staining with CD4-FITC and CD28-APC monoclonal antibodies. Illustrative dot plots showing gating of lymphocytes, CD4^{+} T cells and CD28^{null} T cells in samples with >3% (top) and <2% (bottom) circulating CD28^{null} T cells. The frequency of CD28^{null} T cells was calculated as percentage of CD4^{+} T cells (details in Supplemental Methods). **(B)** Peripheral blood mononuclear cells from ACS patients were cultured alone (w/o, top) or in the presence IL-7 (middle) or IL-15 (bottom) for 4 days and stained with CD4-FITC, CD28-APC, CD14-PE and 7-AAD. Illustrative dot plots showing the gating strategy: following gating of lymphocytes, CD4^{+} T cells and exclusion of dead cells (live CD4^{+} T cells), CD28 expression was used to gate CD28^{null} T cells. **(C)** CD4^{+} T cells from ACS patients were cultured alone (w/o, top) or in the presence IL-7 (middle) or IL-15 (bottom) for 4 days and stained with CD4-FITC, CD28-APC, CD14-PE and 7AAD. Illustrative dot plots showing the gating strategy: following gating of lymphocytes, CD4^{+} T cells and exclusion of dead cells, CD28 expression was used to gate CD28^{null} T cells. Data included in panels **A**, **B**, **C** is representative of more than n=10 different ACS patients

Supplemental Figure 2. Effect of inflammatory cytokines on CD28^{null} T cell expansion. Peripheral blood mononuclear cells from ACS patients with <2% circulating CD28^{null} T cells were cultured alone or in the presence of **(A)** TNF-α (n=8), **(B)** IL-1β (n=6) or **(C)** IL-6 (n=7) as indicated for up to 7 days. Illustrative dot plots show the percentage of CD28^{null} T cells in the analysed samples. Graphs show the percentage of

$CD28^{null}$ T cells for each cytokine and time point. No significant differences were identified (one-way ANOVA with post-test Bonferroni for multiple comparisons)

Supplemental Figure 3. Effect of inflammatory cytokines on $CD28^{null}$ T cell expansion. $CD4^+$ T cells from ACS patients with <2% circulating $CD28^{null}$ T cells were cultured alone or in the presence of the indicated concentrations of **(A)** TNF-α (n=7), **(B)** IL-1β (n=6) or **(C)** IL-6 (n=6) for up to 7 days. Illustrative dot plots show the percentage of $CD28^{null}$ T cells in each sample. Graphs show the percentage of $CD28^{null}$ T cells for each time point. No significant differences were identified (one-way ANOVA with post-test Bonferroni for multiple comparisons)

Supplemental Figure 4. Effect of inflammatory cytokine combinations on $CD28^{null}$ T cell expansion. Peripheral blood mononuclear cells from ACS patients (n=5) with <2% circulating $CD28^{null}$ T cells were cultured alone (w/o) or in the presence of different combinations of inflammatory cytokines (10 ng/ml TNF-α, 10 ng/ml IL-1β, 100 ng/ml IL-6) as indicated for up to 7 days. **(A)** Illustrative dot plots show the percentage of $CD28^{null}$ T cells in each sample. **(B)** Graphs show the percentage of $CD28^{null}$ T cells for each time point (mean±SEM)

Supplemental Figure 5. Effect of inflammatory cytokines on $CD28^{null}$ T cell expansion. PBMCs **(A-C**, n=6**)** or $CD4^+$ T cells **(D-F**, n=6**)** from ACS patients with >3% circulating $CD28^{null}$ T cells were cultured alone (w/o) or in the presence of **(A,D)** TNF-α, **(B,E)** IL-1β or **(C,F)** IL-6 as indicated for up to 7 days. Illustrative dot plots show the percentage of $CD28^{null}$ T cells in the analysed samples. Graphs show the percentage of $CD28^{null}$ T cells for each cytokine and time point. No significant differences were identified (one-way ANOVA with post-test Bonferroni for multiple comparisons)

Supplemental Figure 6. Effect of different concentrations of IL-7 and IL-15 on CD28^{null} T cell expansion at different time points. Peripheral blood mononuclear cells (**A-D** n=7) or CD4^{+} T cells (**E,F** n=7) from ACS patients with >3% circulating CD28^{null} T cells were treated with 10 ng/ml or 50 ng/ml IL-7 or IL-15 for up to 7 days. **(A,C,E)** Illustrative dot plots show the percentage of CD28^{null} T cells. **(B,D,F)** Graphs show the percentage of CD28^{null} T cells in untreated samples (w/o) or after treatment with IL-7 or IL-15.* p<0.05; ** p<0.01; *** p<0.001; **** p<0.0001 (paired two-tailed Student's t test)

Supplemental Figure 7. Effect of IL-7 and IL-15 on CD28^{null} T cell expansion. Peripheral blood mononuclear cells (n=10) from ACS patients with <2% circulating CD28^{null} T cells were treated with 10 ng/ml or 50 ng/ml IL-7 or IL-15 for 4 and 7 days. **(A,C)** Illustrative dot plots display CD28^{null} T cell percentage following **(A)** IL-7 and **(C)** IL-15 treatment at the indicated time points. **(B,D)** Graphs show CD28^{null} T cell percentage in untreated samples (w/o) or after treatment with the indicated concentrations of **(B)** IL-7 or **(D)** IL-15. * p<0.05; ** p<0.01; ns, not significant (paired two-tailed Student's t test)

Supplemental Figure 8. Effect of IL-7 and IL-15 on CD28^{null} T cell expansion. CD4^{+} T cells from ACS patients with <2% CD28^{null} T cells were treated with 10 ng/ml or 50 ng/ml IL-7 or IL-15 for 4 (n=8), 7 (n=8) or 11 (n=6) days. **(A,C)** Illustrative dot plots show the percentage of CD28^{null} T cells following **(A)** IL-7 and **(C)** IL-15 treatment at the indicated time points. Graphs display the percentage of CD28^{null} T cells in untreated samples (w/o) or after treatment with the indicated concentration of **(B)** IL-7 or **(D)** IL-15. * p<0.05; ** p<0.01; ns, not significant (paired two-tailed Student's t test)

Supplemental Figure 9. Effect of inflammatory cytokines on CD28^{null} T cell activation. CD4^{+} T cells from ACS patients (n=6) were cultured alone (w/o) or treated with 10 ng/m TNF-α, 10 ng/m IL-1β, or 100 ng/m IL-6 for 4 days. The activation markers

CD69 and HLA-DR were analysed on CD28^{null} and CD28^{pos} T cells. Graphs show the percentage of CD69^{+} cells **(A)** or HLA-DR^{+} cells **(B)** in cytokine-treated and untreated samples. No significant differences were identified (two-tailed Wilcoxon matched-pairs signed rank test)

Supplemental Figure 10. Effect of IL-7 and IL-15 on expression of chemokine receptors by CD28^{null} T cells. CD4^{+} T cells from ACS patients (n=10) were cultured alone (w/o) or treated with 50 ng/ml IL-7 or IL-15 for 3-4 days. Expression of CCR5 and CXCR3 was analysed on CD28^{null} and CD28^{pos} T cells. Illustrative dot plots and graphs display the percentage of CCR5^{+} cells **(A,B)** and CXCR3^{+} cells **(C,D)** in CD28^{null} and CD28^{pos} T cells; dashed gates, isotype control antibody (Ctrl). ** p<0.01 (two-tailed Wilcoxon matched-pairs signed rank test)

Supplemental Figure 11. Effect of IL-7 and IL-15 on expression of memory markers by CD28^{null} T cells. CD4^{+} T cells from ACS patients (n=10) were cultured alone (w/o) or treated with 50 ng/ml IL-7 or IL-15 for 3-4 days. Expression of CD62L, CCR7, CD45RA and CD45RO was analysed on CD28^{null} and CD28^{pos} T cells. Illustrative dot plots and graphs display the percentage of CD45RA^{+}CD62L^{+}, CD45RA^{-}CD62L^{+}, CD45RA^{-}CD62L^{-} and CD45RA^{+}CD62L^{-} cells **(A,B)**; CD45RA^{+}CCR7^{+}, CD45RA^{-}CCR7^{+}, CD45RA^{-}CCR7^{-} and CD45RA^{+}CCR7^{-} cells **(C,D)**; and CD45RA^{+}CD45RO^{-}, CD45RA^{+}CD45RO^{+} and CD45RA^{-}CD45RO^{+} cells **(E,F)** in CD28^{null} and CD28^{pos} T cells (mean±SEM); dashed gates, isotype control antibody (Ctrl). No significant differences were identified (two-way ANOVA with post-test Bonferroni for multiple comparisons)

Supplemental Figure 12. Comparison of different concentrations of IL-7 or IL-15 on CD28^{null} T cell proliferation at different time points. CD4^{+} T cells from ACS patients (n=5) were labelled with CFSE and cultured in the presence of 10 or 50 ng/ml IL-7 **(A)** or IL-15 **(B)** up to 7 days. Graphs show proliferation of CD28^{null} and CD28^{pos} T cells in

untreated samples (w/o) and after cytokine treatment at the indicated time points. * $p<0.05$; ** $p<0.01$; *** $p<0.001$; **** $p<0.0001$; ns, not significant (paired two-tailed Student's t test)

Supplemental Figure 13. Cytokine plasma levels in ACS patients. Cytokine levels were quantified in EDTA-plasma samples from ACS patients with >3% circulating $CD28^{null}$ T cells (with $CD28^{null}$ T cell expansion; n=38) and ACS patients with <2% circulating $CD28^{null}$ T cells (without $CD28^{null}$ T cell expansion; n=38). Graphs show the concentration of the indicated cytokines in the two study groups (**A.** IL-7 and IL-15; **B.** TNF-α, IL-1β and IL-6). The horizontal bar indicates the mean concentration. No significant differences were identified (two-tailed Mann-Whitney test)

Supplemental Figure 1

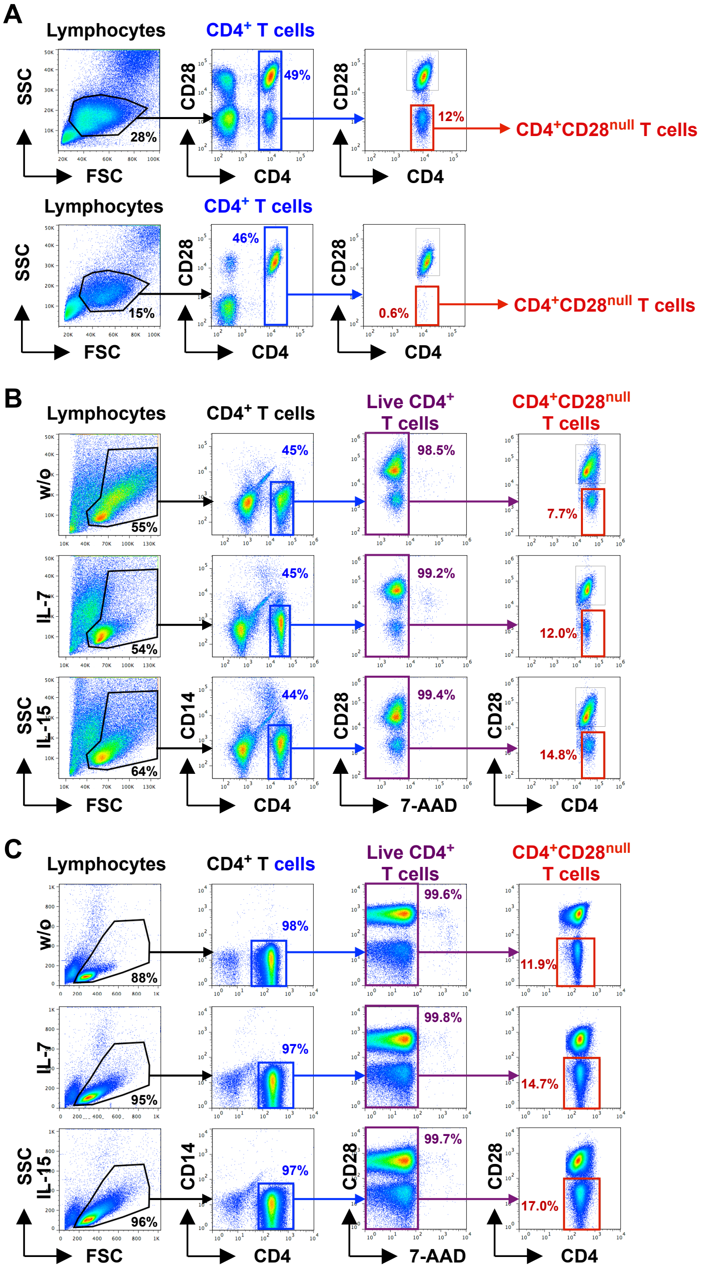

Supplemental Figure 2

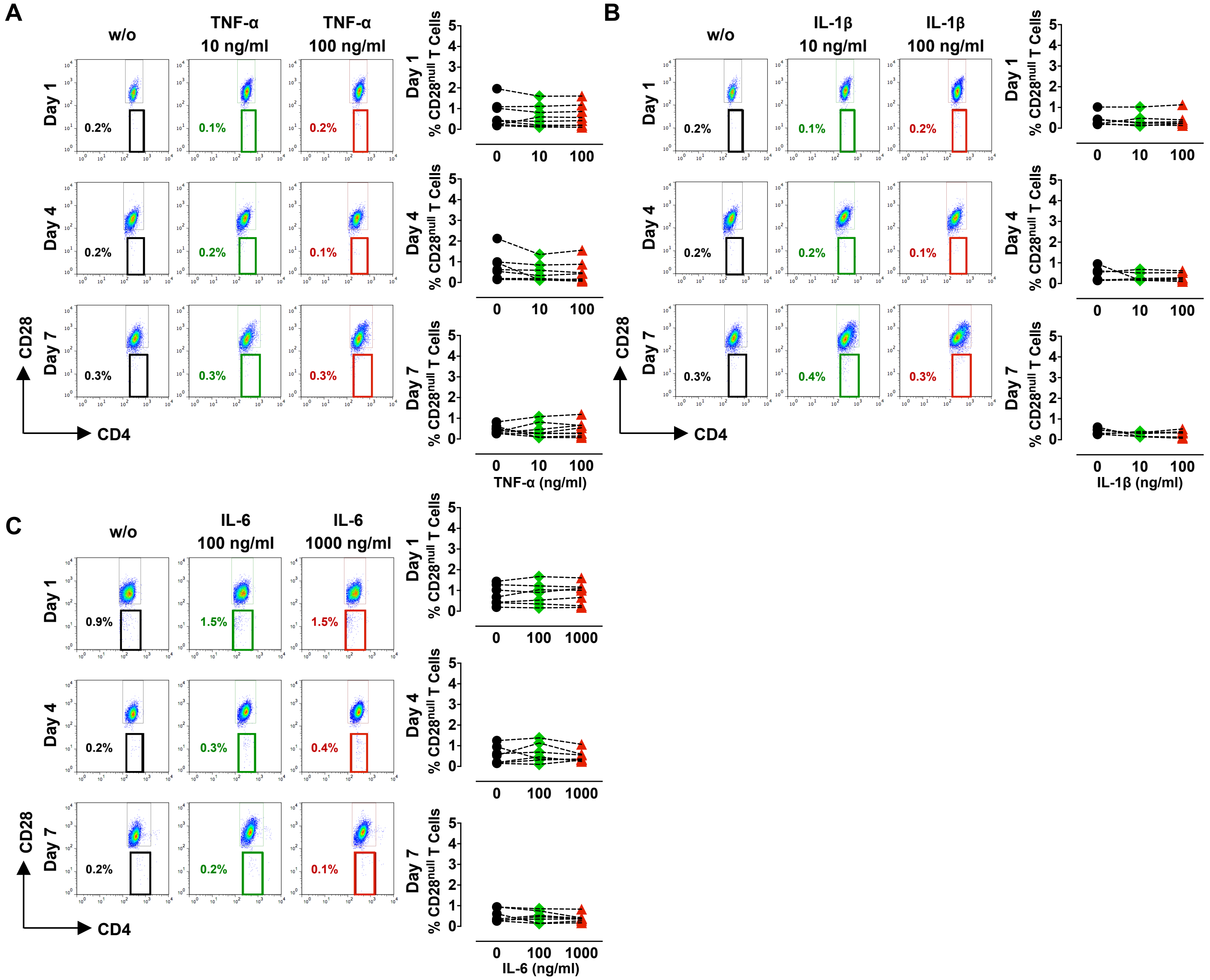

Supplemental Figure 3

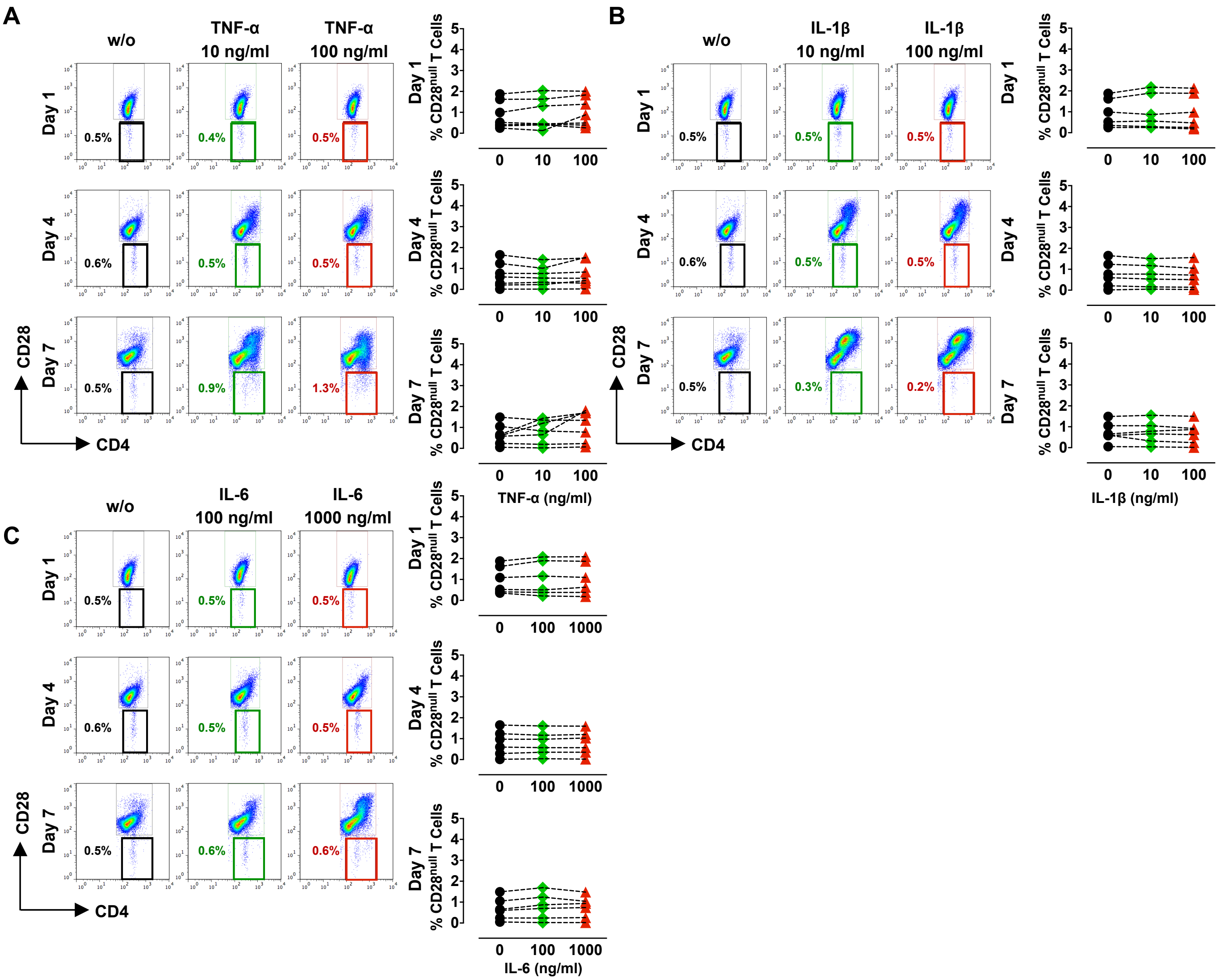

Supplemental Figure 4

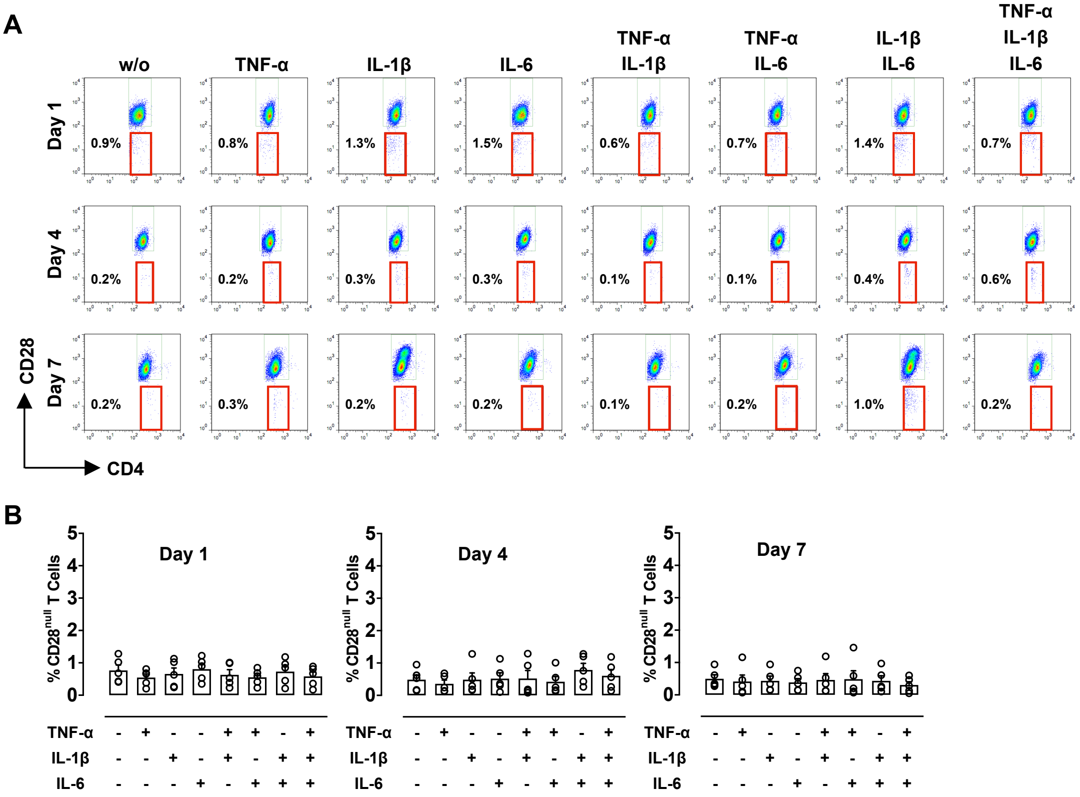

Supplemental Figure 5

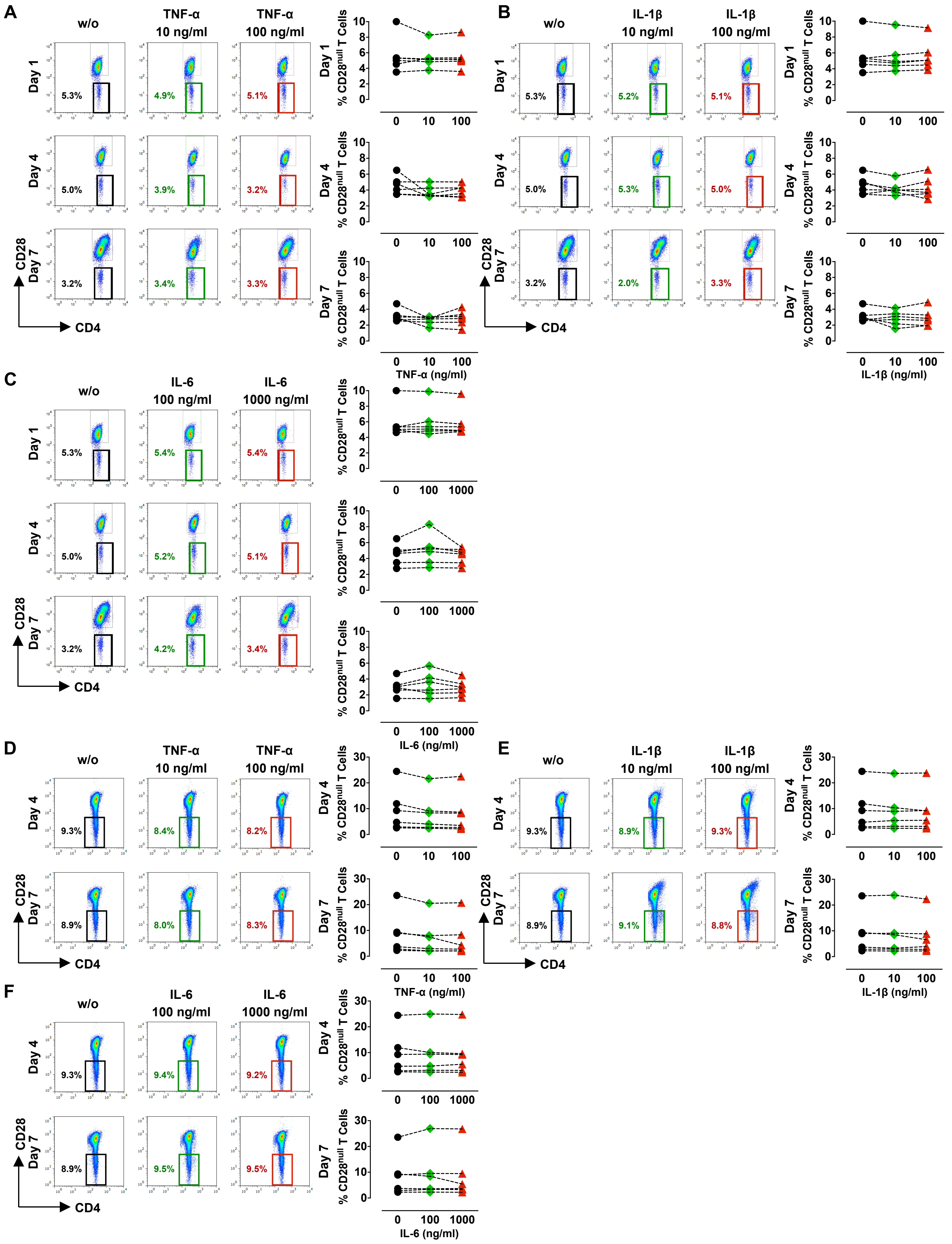

Supplemental Figure 6

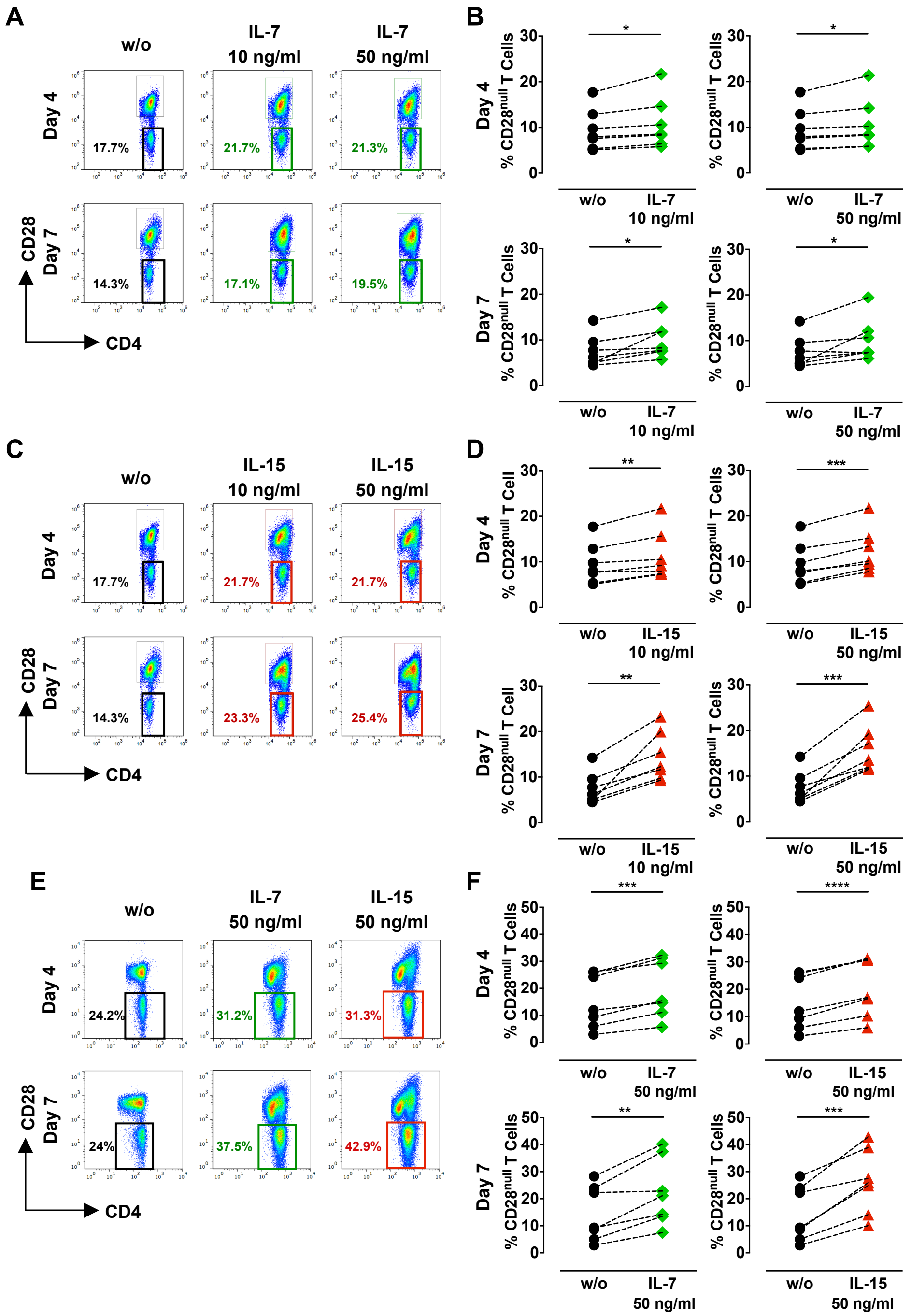

Supplemental Figure 7

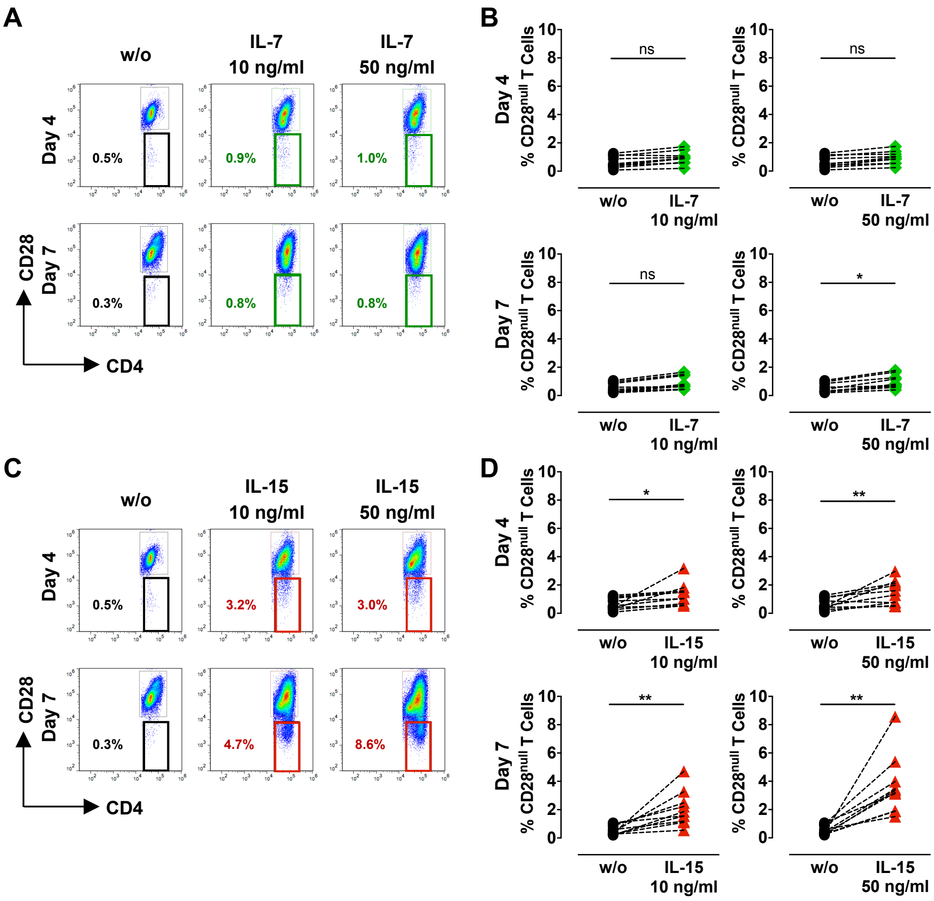

Supplemental Figure 8

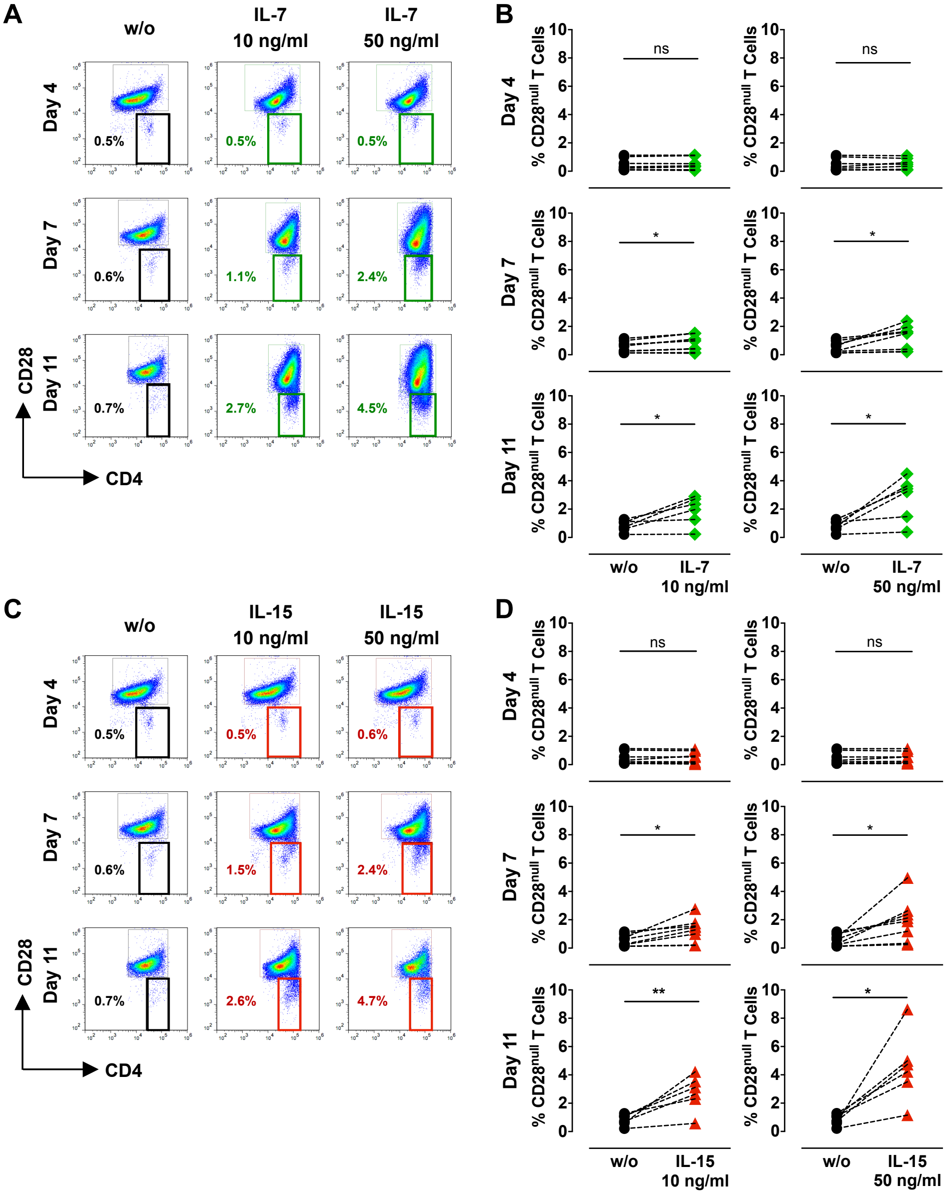

Supplemental Figure 9

A

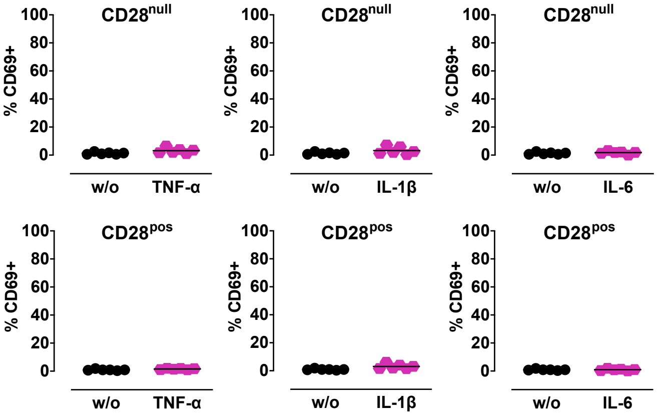

B

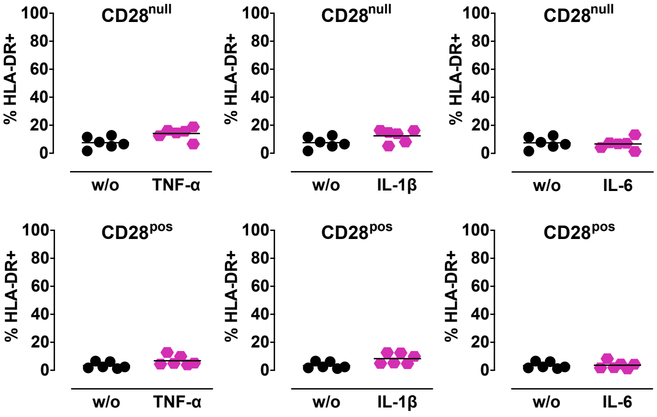

Supplemental Figure 10

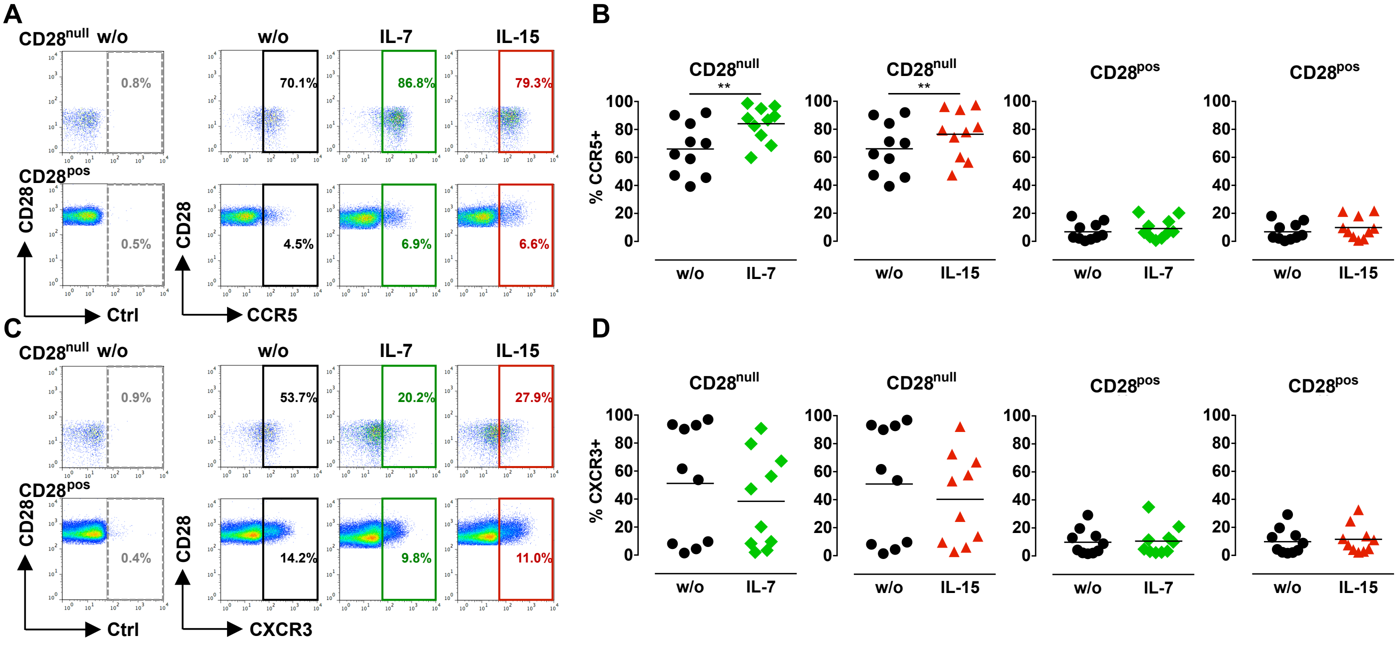

Supplemental Figure 11

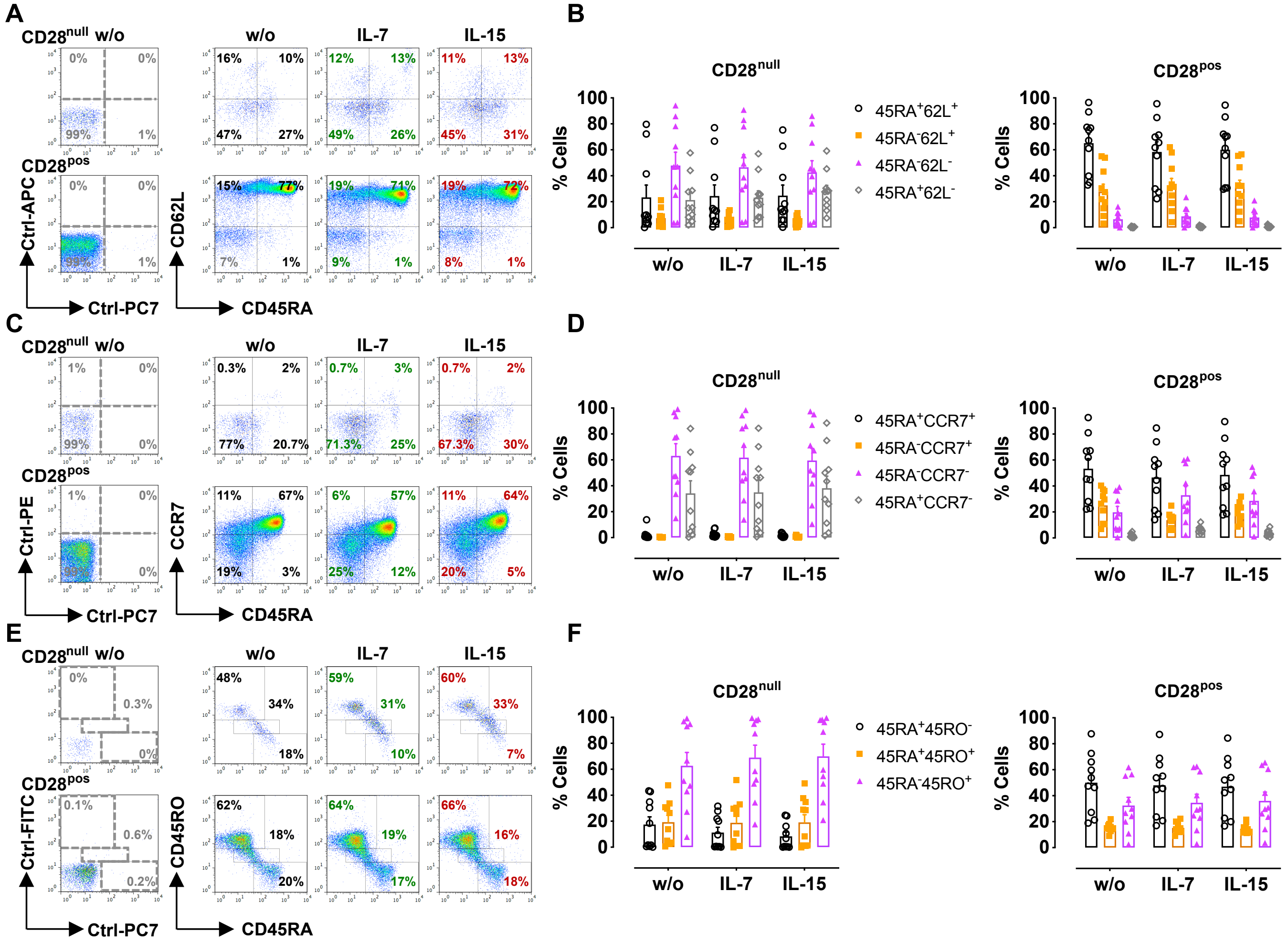

Supplemental Figure 12

A

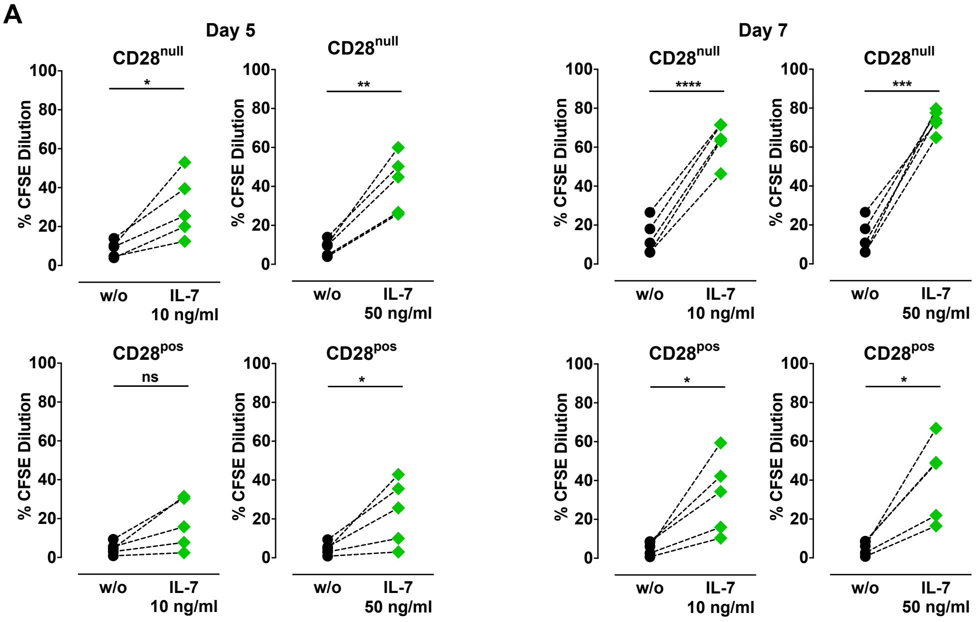

B

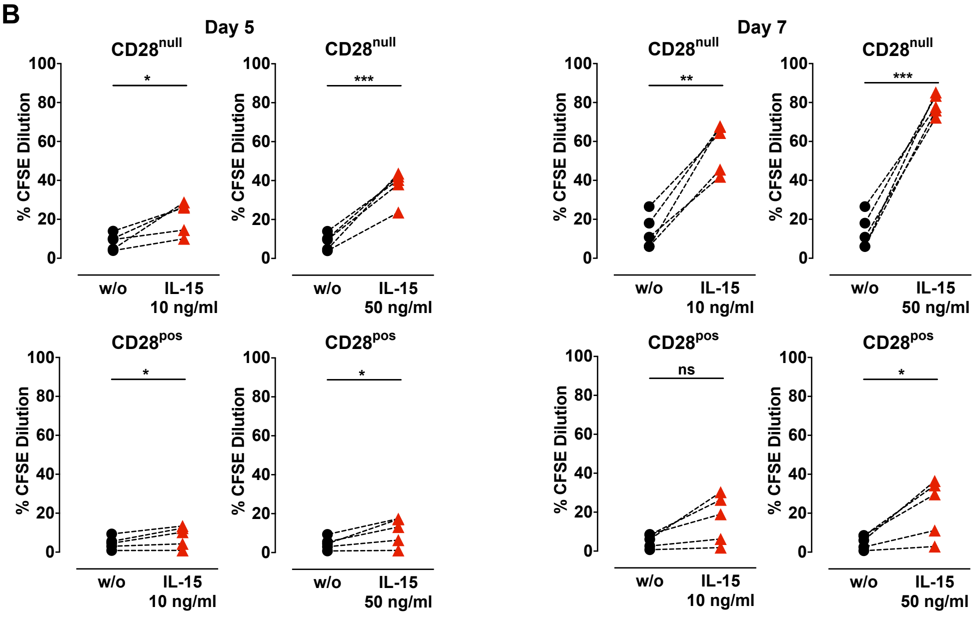

Supplemental Figure 13

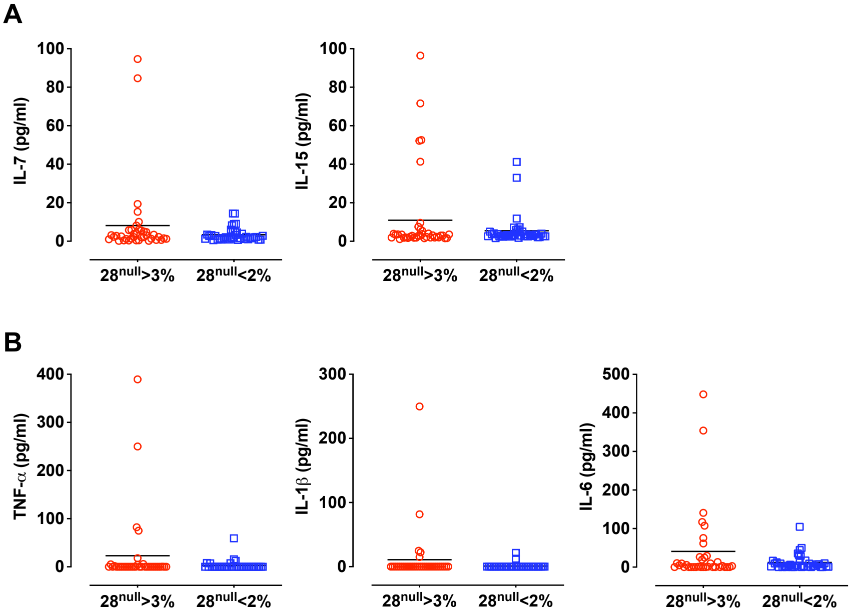

Supplement: cvaa202_Supplementary_Data [file cvaa202_supplementary_data.pdf]
